# Eco-conscious Fe–Cu bimetallic nanozymes as a multimodal colorimetric platform for glucose and hexavalent chromium determination

**DOI:** 10.1039/d6ra07383b

**Published:** 2026-09-29

**Authors:** Saja A. Althobaiti, Ola G. Hussein

**Affiliations:** a Department of Chemistry, College of Science and Humanities in Al-Kharj, Prince Sattam Bin Abdulaziz University Al-Kharj 11942 Saudi Arabia; b Department of Pharmaceutical Chemistry, Faculty of Pharmacy, Future University in Egypt Cairo 11835 Egypt ola.farag@fue.edu.eg

## Abstract

The integration of sustainable nanotechnology with portable analytical systems offers a promising strategy for advancing point-of-care (POC) diagnostic platforms with Green and White Analytical Chemistry. In this work, eco-conscious iron–copper bimetallic nanozymes (Fe–Cu BNPs) were synthesized through a facile aqueous approach and explored as multifunctional peroxidase-mimicking catalysts for biomedical and environmental sensing applications. The synthesized Fe–Cu BNPs functioned as highly active catalytic interfaces converting 3,3′,5,5′-tetramethylbenzidine (TMB) into a blue oxidized product through a hydrogen peroxide-dependent reaction, thereby enabling sensitive colorimetric determination. The developed platform exhibited good analytical performance with linear ranges of 10–700 µM for H_2_O_2_, 10–500 µM for glucose, and 0–500 µM for Cr(vi) (*R*^2^ > 0.99). The sensor demonstrated high sensitivity, favorable selectivity, and successful application to human serum samples. Kinetic analysis revealed a low *K*_m_ value (0.18 mM) and a *V*_max_ of 8.92 × 10^−8^ M s^−1^ confirming the favorable catalytic efficiency of the Fe–Cu BNP nanozymes. To facilitate portable monitoring, a multimodal sensing platform was developed by integrating spectrophotometric measurements with smartphone-assisted digital image colorimetry and Arduino-enabled data acquisition. The analytical response was monitored by capturing the developed colorimetric signal with a smartphone camera followed by quantitative RGB image analysis allowing convenient point-of-care glucose determination without reliance on laboratory-based instrumentation. The fabricated sensing system displayed effective catalytic performance, an extensive linear working range, high analytical sensitivity, low limits of detection, and good resistance to interference from commonly encountered biomolecules. The practical applicability of the proposed methodology was demonstrated through successful glucose determination in human serum samples while the Fe–Cu BNP platform exhibited promising analytical performance for Cr(vi) detection. Mechanistic studies suggested that the synergistic interaction between iron and copper active centers promotes electron-transfer processes and reactive oxygen species generation resulting in enhanced catalytic activity. Furthermore, sustainability assessment using several assessment tools confirming the environmental compatibility, analytical reliability, and practical utility of the developed platform. Overall, this work presents a sustainable multifunctional Fe–Cu nanozyme system that combines eco-conscious aqueous synthesis, portable digital sensing, and comprehensive sustainability evaluation within a unified analytical framework. The developed strategy provides a promising platform for next-generation point-of-care diagnostics and environmental monitoring.

## Abstract 2

The integration of sustainable nanotechnology with portable analytical systems offers a promising strategy for advancing point-of-care (POC) diagnostic platforms aligned with the principles of Green and White Analytical Chemistry. Graphical abstract design assisted with ChatGPT.

## Graphical abstract

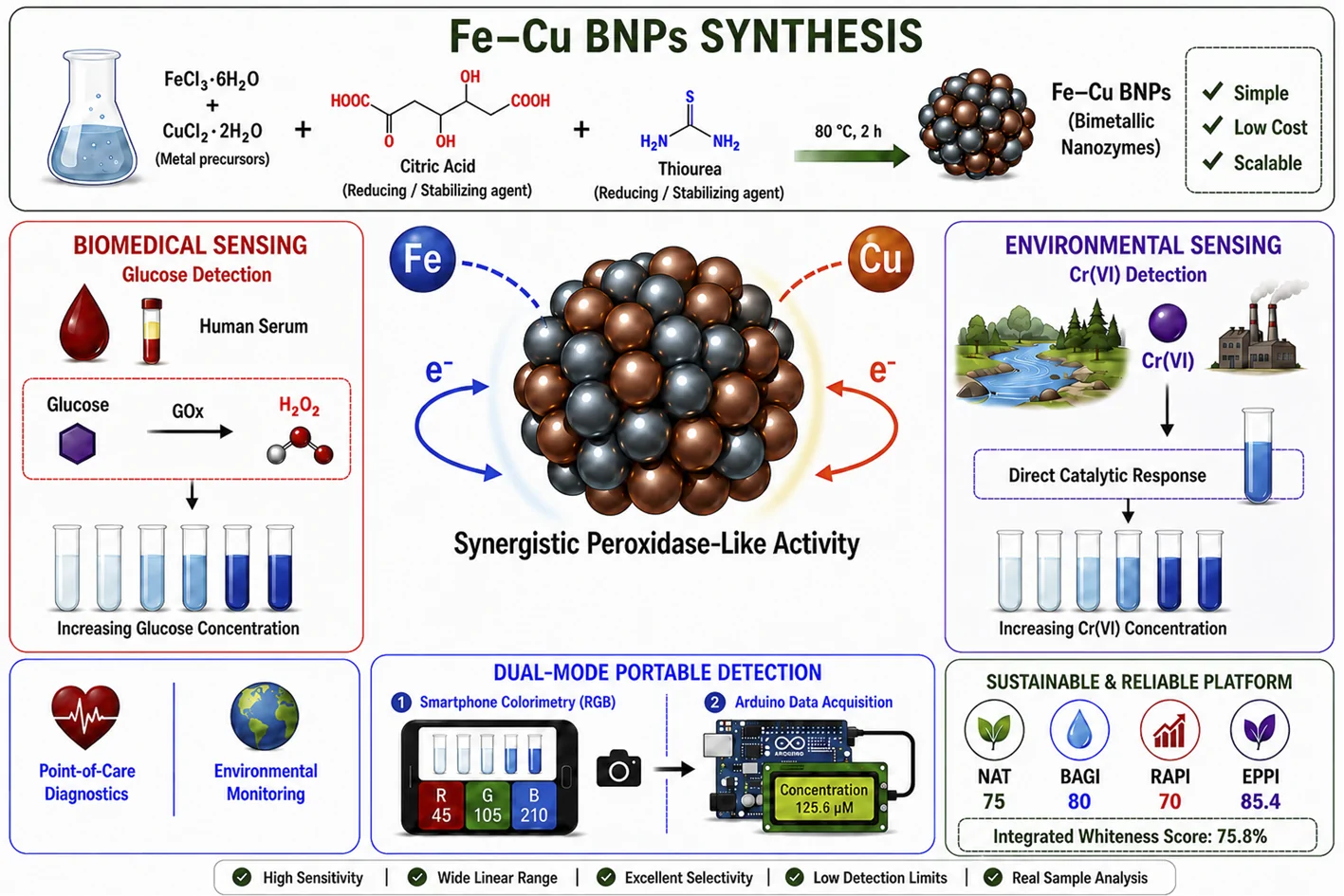

## 1. Introduction

Growing interest in fast, reliable, and affordable biochemical sensing has accelerated the development of point-of-care (POC) diagnostic platforms especially for the detection and monitoring of clinically important biomarkers such as glucose.^1–3^ Glucose determination remains one of the most critical analytical tasks in clinical chemistry due to its direct association with diabetes mellitus, a chronic metabolic disorder affecting a large global population.^4,5^ Despite their high analytical accuracy, conventional glucose assays typically depend on sophisticated laboratory facilities, costly analytical equipment, and skilled operators restricting their implementation in resource-constrained settings and continuous point-of-care health monitoring.^6^ These limitations have encouraged the development of alternative sensing strategies that are portable, simple, and capable of delivering rapid analytical responses.^7^

In recent years, nanozyme-based catalytic systems have emerged as a powerful substitute for natural enzymes in biosensing applications. Nanozymes, defined as nanomaterials with enzyme-like catalytic properties offer several advantages over natural enzymes including superior stability, lower cost, tunable activity, and resistance to harsh environmental conditions.^8–18^ Among various nanozyme activities, peroxidase-mimicking behavior has attracted considerable attention due to its wide application in colorimetric biosensing platforms. These nanozymes typically catalyze chromogenic substrates such as 3,3′,5,5′-tetramethylbenzidine (TMB) in the presence of hydrogen peroxide producing a visually detectable color change that can be easily quantified.^19–21^

Bimetallic nanozymes have recently gained prominence due to their synergistic electronic interactions between two metal components which often result in enhanced catalytic activity compared to their monometallic counterparts.^22,23^ In particular, iron- and copper-based nanostructures have demonstrated promising catalytic performance owing to their variable oxidation states and strong redox cycling capabilities.^24,25^ The combination of iron and copper within a single nanostructure can facilitate efficient electron transfer processes and promote the generation of reactive oxygen species, thereby significantly improving peroxidase-like catalytic efficiency.^26,27^ Despite these advantages, the development of environmentally benign synthesis routes for such bimetallic systems remains a key challenge in sustainable nanotechnology.

Eco-friendly synthetic strategies have gained considerable attention as sustainable substitutes for traditional chemical and physical nanoparticle production techniques.^28,29^ Environmentally benign synthetic strategies commonly employ naturally occurring biomolecules, plant-based metabolites, or other sustainable reducing and capping agents to facilitate nanoparticle formation under mild reaction conditions. By avoiding hazardous reagents and energy-intensive processes these approaches minimize environmental burden while simultaneously improving the biocompatibility and surface characteristics of the synthesized nanomaterials thereby increasing their suitability for diverse biomedical applications.^30,31^ Integrating green chemistry principles into nanozyme fabrication therefore represents a critical step toward sustainable diagnostic technology development.^32^ Furthermore, such approaches strongly support the objectives of White Analytical Chemistry which seeks to balance analytical performance, environmental responsibility, and practical usability within a unified framework.^33–35^

In addition to biomedical diagnostics, growing concerns regarding environmental pollution have intensified the need for sensitive and reliable monitoring technologies for hazardous contaminants.^36,37^ Among various toxic pollutants, hexavalent chromium [Cr(vi)] is widely regarded as a highly toxic and environmentally concerning heavy metal contaminant due to its high toxicity, carcinogenicity, and persistence in aquatic ecosystems.^38,39^ Cr(vi) is widely released from industrial activities including electroplating, leather tanning, pigment production, and metal finishing processes posing significant risks to both environmental and human health.^40^ Consequently, the development of simple, rapid, and cost-effective analytical methods for Cr(vi) determination has become an important objective in environmental analysis.^41^ Nanozyme-based sensing systems have attracted considerable attention for heavy-metal detection because of their exceptional catalytic performance and adaptable surface characteristics enabling highly sensitive analysis through simple and efficient detection strategies that overcome many limitations of conventional analytical methods.^42,43^

Alongside the rapid development of advanced nanomaterials, the incorporation of digital technologies including smartphone-based platforms and microcontroller-assisted devices has significantly transformed modern analytical sensing methodologies. Smartphone-based colorimetric analysis in particular has gained widespread attention due to the prevalence, portability, and high-resolution imaging capabilities of modern mobile devices.^44^ By converting color intensity changes into quantitative digital signals through image processing techniques such as RGB analysis, smartphones can serve as powerful analytical tools for decentralized diagnostics.^45,46^ Furthermore, the incorporation of microcontrollers such as Arduino enables automation, signal processing, and real-time data acquisition, thereby enhancing the precision and reproducibility of portable sensing systems.^47^

The combination of nanozyme catalysis with smartphone-assisted and Arduino-integrated detection platforms offers a promising route toward fully portable and user-friendly biosensing devices. Such hybrid systems enable multimodal analysis where conventional spectrophotometric measurements can be complemented with digital image-based quantification ensuring higher analytical reliability and broader applicability. This multimodal detection strategy enhances analytical flexibility while maintaining operational simplicity making it suitable for point-of-care biomedical analysis and potential field-deployable environmental sensing applications.

In the present work, Fe–Cu bimetallic nanozymes synthesized through an eco-conscious aqueous approach were explored as peroxidase-mimicking catalysts for versatile analytical sensing applications. The proposed sensing platform combines glucose oxidase-mediated enzymatic reactions with Fe–Cu nanozyme-catalyzed color development for sensitive glucose determination. In addition, the sensing capability of the synthesized nanozymes was extended toward the colorimetric determination of hexavalent chromium [Cr(vi)] demonstrating their potential for environmental monitoring. To improve accessibility and field applicability, a multimodal detection strategy integrating conventional spectrophotometry, smartphone-assisted digital image colorimetry, and Arduino-based signal acquisition was developed. Furthermore, the cooperative contribution of Fe and Cu to the peroxidase-like catalytic activity was investigated through comparative experiments involving Fe-only NPs, Cu-only NPs, a physical Fe/Cu mixture, and the synthesized Fe–Cu BNPs, while reactive oxygen species generation was evaluated by EPR spin-trapping analysis.

The novelty of the present work lies in integrating an eco-consciously synthesized Fe–Cu bimetallic nanozyme with multifunctional colorimetric sensing, mechanistic investigation, portable digital detection, and comprehensive sustainability assessment within a single analytical platform. In contrast to conventional nanozyme-based approaches primarily focused on individual analytes or laboratory-based detection, the developed platform combines Fe–Cu BNP-mediated H_2_O_2_ sensing, a GOx-coupled cascade for glucose determination, and colorimetric detection of Cr(vi). Comparative catalytic controls and EPR spin-trapping analysis provide experimental support for the proposed cooperative Fe–Cu catalytic contribution and ROS involvement in the peroxidase-like reaction. Furthermore, smartphone-assisted image analysis and Arduino-based signal acquisition provide complementary portable detection modes reducing dependence on conventional laboratory instrumentation. A further distinctive feature is the comprehensive sustainability assessment using NAT, EPPI, BAGI, and RAPI metrics integrating nanomaterial sustainability, environmental performance, practical applicability, and analytical performance. Collectively, these features highlight the significance of the proposed platform as a versatile approach for decentralized biomedical analysis and potential environmental sensing applications.

## 2. Experimental

### 2.1. Materials and reagents

All substances employed throughout the investigation were of high analytical purity and were utilized as received without any additional treatment or purification steps. Ultrapure double-distilled water obtained from an ELGA PURELAB Flex water purification system was employed throughout the study as the reaction medium and solvent to ensure high purity and reduce the risk of contamination during experimental procedures. Iron and copper precursor salts required for the synthesis of Fe–Cu bimetallic nanoparticles (Fe–Cu BNPs) were selected based on their high solubility and suitability for the synthesis approaches. Hydrogen peroxide (H_2_O_2_), 3,3′,5,5′-tetramethylbenzidine (TMB), glucose oxidase (GOx), hydrochloric acid (HCl), potassium dichromate (K_2_Cr_2_O_7_) as a source of hexavalent chromium [Cr(vi)], citric acid, thiourea, sodium acetate, and acetic acid, *tert*-butyl hydroperoxide (TBHP), persulfate (S_2_O_8_^2−^), and hypochlorite (ClO^−^) were purchased from Sigma-Aldrich (Germany).

Citric acid and thiourea were employed as reducing and stabilizing agents during the aqueous synthesis of the Fe–Cu bimetallic nanostructures. Although the aqueous medium and relatively mild reaction conditions support a more environmentally considerate synthesis, the safety and environmental aspects associated with thiourea were considered in the overall sustainability assessment. Fresh buffer solutions were prepared as required for each experimental set. Acetate buffers were prepared using sodium acetate and acetic acid. A 0.2 M acetate buffer (pH 4.0) was used as the optimized medium for the Fe–Cu BNP/TMB catalytic reaction, whereas a 50 mM acetate buffer (pH 5.1) was employed for the GOx-mediated glucose oxidation step. The required pH values were verified and adjusted as necessary before use.

### 2.2. Instrumentation and data analysis

The physicochemical, morphological, and catalytic characteristics of the prepared iron–copper bimetallic nanoparticles (Fe–Cu BNPs) were investigated using several complementary characterization techniques. The surface architecture and nanoscale morphology were evaluated by field-emission scanning electron microscopy (Quanta FEG-250) and high-resolution transmission electron microscopy (JEM-2100 HRT, JEOL, Japan), whereas elemental distribution and composition were verified using energy-dispersive X-ray spectroscopy (EDX). The optical features and catalytic response of the Fe–Cu BNPs were monitored using a Shimadzu UV-1900i PC UV-Visible spectrophotometer with a spectral bandwidth of 1 nm and a scanning rate of 2800 nm min^−1^. Surface functional moieties were examined by Fourier transform infrared spectroscopy (FTIR; Shimadzu IR-435), while crystalline structure and phase characteristics were assessed through X-ray diffraction using a Rigaku SmartLab diffractometer. X-ray photoelectron spectroscopy (XPS) was employed to investigate the surface chemical states of Fe and Cu in the synthesized Fe–Cu BNPs. XPS measurements were performed using a monochromatic Al Kα X-ray source (*hν* = 1486.6 eV). High-resolution Fe 2p and Cu 2p spectra were acquired and deconvoluted to identify the corresponding oxidation states and satellite features. Binding energies were referenced to the C 1s peak at 284.8 eV. Peak fitting and background correction were applied to resolve the individual contributions of the different Fe and Cu chemical states. Electron paramagnetic resonance (EPR) spectroscopy was employed to investigate reactive oxygen species generated during the Fe–Cu BNP-mediated catalytic reaction using 5,5-dimethyl-1-pyrroline *N*-oxide (DMPO) as a spin-trapping agent. EPR measurements were performed under appropriate acquisition conditions to detect and characterize the generated radical species.

For portable colorimetric analysis, digital images were acquired using an iPhone 15 Pro smartphone (Apple, USA) and processed using ImageJ software (National Institutes of Health, Maryland, USA). Arduino-assisted measurements were performed using a TCS34725 RGB color sensor connected to an Arduino Nano microcontroller through an I^2^C interface. The TCS34725 sensor contains integrated photodiodes for measurement of the red, green, blue, and clear light components and an on-chip analog-to-digital converter for conversion of the detected optical signals into digital values. The environmental sustainability and analytical performance of the developed sensing platform were systematically evaluated using web-based assessment tools including the Environmental Performance-Practicality Index (EPPI), Blue Applicability Grade Index (BAGI), Red Analytical Performance Index (RAPI), and NanoMaterial Assessment Tool (NAT).

### 2.3. Synthesis of Fe–Cu bimetallic nanoparticles

Fe–Cu bimetallic nanoparticles (Fe–Cu BNPs) were synthesized through a facile aqueous reduction method using iron(iii) chloride hexahydrate (FeCl_3_·6H_2_O) and copper(ii) chloride dihydrate (CuCl_2_·2H_2_O) as the Fe and Cu precursors, respectively. Briefly, 2.0 mmol of FeCl_3_·6H_2_O and 1.0 mmol of CuCl_2_·2H_2_O were separately dissolved in 25 mL of double-distilled water. The two solutions were then mixed to obtain a Fe/Cu molar ratio of 2 : 1 and continuously stirred for 15 min. Subsequently, 5.0 mmol of citric acid dissolved in 10 mL of double-distilled water was added dropwise to the metal-ion mixture under vigorous stirring, followed by the gradual addition of 5.0 mmol of thiourea dissolved in 10 mL of double-distilled water.

The resulting mixture was continuously stirred and maintained at 80 °C for 2 h to promote the formation of the Fe–Cu bimetallic nanostructures. During the reaction, a gradual color change was observed indicating nanoparticle formation. The reaction time was fixed at 2 h, after which no further visible change in the color or appearance of the reaction mixture was observed; this predetermined reaction time was therefore used as the operational endpoint of the synthesis.

Following the 2 h reaction period, the mixture was allowed to cool naturally to room temperature. The formed Fe–Cu BNPs were recovered by centrifugation at 10 000 rpm for 15 min. The precipitate was collected and washed three times with double-distilled water followed by two washes with ethanol to remove unreacted precursors and weakly adsorbed organic species. Finally, the purified nanoparticles were dried at 60 °C for 12 h and stored in airtight containers at room temperature until further characterization and catalytic experiments.

### 2.4. Investigation of the prepared Fe–Cu bimetallic nanostructures

A series of analytical techniques was employed to gain insight into the physicochemical characteristics of the fabricated Fe–Cu bimetallic nanoparticles. The external appearance, particle architecture, and nanoscale features were visualized through scanning and transmission electron microscopy. In addition, elemental analysis was carried out using energy-dispersive X-ray measurements to verify the incorporation of both metallic constituents and assess their spatial distribution within the synthesized nanomaterial. FTIR spectroscopy was performed to identify surface functional groups and investigate the interaction of stabilizing agents with the nanoparticle surface. The crystalline structure of the synthesized nanozymes was examined using XRD analysis. In addition, UV-Vis spectroscopy was utilized to evaluate optical properties and monitor the catalytic oxidation of TMB. These analyses were conducted to verify the successful synthesis of Fe–Cu BNPs and their suitability for glucose sensing and Cr(vi) detection applications.

### 2.5. Investigation of the catalytic activity of Fe–Cu BNPs

The peroxidase-like catalytic activity of the synthesized Fe–Cu BNPs was evaluated through the catalytic oxidation of 3,3′,5,5′-tetramethylbenzidine (TMB) in the presence of hydrogen peroxide. The reaction mixture was prepared by mixing 500 µL of Fe–Cu BNP suspension (1.0 mg mL^−1^), 500 µL of TMB solution (5.0 mM), and 200 µL of H_2_O_2_ solution (10 mM) with 1.8 mL of 0.2 M acetate buffer (pH 4.0) giving a final reaction volume of 3.0 mL. The reaction mixture was incubated at 40 °C for 10 min. The absorption spectrum was subsequently recorded over the wavelength range of 300.0–800.0 nm and the absorbance of oxidized TMB (oxTMB) was measured at 652.0 nm. To verify the catalytic role of the prepared nanomaterial, parallel assays were performed in the absence of Fe–Cu BNPs. Under identical experimental conditions, no significant optical response was observed demonstrating that the color-forming reaction was dependent on the catalytic activity of the synthesized bimetallic nanozyme.

To investigate the cooperative catalytic contribution of Fe and Cu, comparative control experiments were performed using Fe-only NPs, Cu-only NPs, a physical mixture of the separately prepared Fe- and Cu-based materials and the synthesized Fe–Cu BNPs. The Fe-only and Cu-only materials were prepared using the corresponding individual metal precursor under otherwise comparable synthesis conditions, while the physical Fe/Cu mixture was obtained by combining the separately prepared monometallic materials. All catalyst systems were evaluated at the same total catalyst loading under identical TMB/H_2_O_2_ reaction conditions. Briefly, 500 µL of catalyst suspension (1.0 mg mL^−1^), 500 µL of TMB solution (5.0 mM), and 200 µL of H_2_O_2_ solution (10 mM) were mixed with 1.8 mL of 0.2 M acetate buffer (pH 4.0), followed by incubation at 40 °C for 10 min. Catalytic activity was evaluated by measuring the absorbance of oxTMB at 652.0 nm. All quantitative measurements were independently performed in triplicate (*n* = 3) using separately prepared reaction mixtures under identical experimental conditions and the results were expressed as mean ± standard deviation (SD).

Reactive oxygen species generated during the Fe–Cu BNP/H_2_O_2_ catalytic reaction were further investigated by EPR spectroscopy using DMPO as a spin-trapping agent. The resulting EPR spectra were examined for radical-adduct signals assigned to hydroxyl- and superoxide-derived species, while a DMPO-containing blank without the Fe–Cu BNP/H_2_O_2_ catalytic system was measured under identical conditions as the control.

### 2.6. Optimization of assay conditions

To achieve maximum catalytic performance, the influence of experimental parameters including pH, temperature, reaction time, and reagent concentrations was systematically evaluated. The catalytic efficiency of the Fe–Cu BNPs was assessed by monitoring the absorbance of oxidized TMB at 652.0 nm. The optimum conditions were selected based on the highest colorimetric response.

#### 2.6.1. Optimization of solution pH for peroxidase-mimicking activity of Fe–Cu BNPs

The effect of solution pH on the peroxidase-like catalytic activity of the synthesized Fe–Cu BNPs was systematically investigated over the pH range of 2.0–8.0 using appropriate buffer systems for the different pH regions. Glycine-HCl buffer was used for pH 2.0–3.0, acetate buffer for pH 4.0–5.8, and phosphate buffer for pH 6.0–8.0. All buffer solutions were prepared at the same concentration (0.2 M) to provide comparable buffer concentrations throughout the pH optimization study. All other experimental parameters were kept constant and the catalytic oxidation of TMB was monitored by measuring the absorbance of oxTMB at 652.0 nm.

#### 2.6.2. Effect of temperature on the catalytic activity of Fe–Cu BNPs

The influence of reaction temperature on the peroxidase-like activity of the synthesized Fe–Cu BNPs was systematically evaluated by conducting the catalytic oxidation of TMB over a temperature range of 20–80 °C. All other experimental variables were maintained constantly throughout the study. The catalytic activity at each temperature was assessed by monitoring the absorbance of oxidized TMB at 652.0 nm.

#### 2.6.3. Effect of reaction time on the catalytic activity of Fe–Cu BNPs

The effect of incubation duration on the peroxidase-like activity of the synthesized Fe–Cu BNPs was systematically investigated by following the catalytic oxidation of TMB over reaction times ranging from 2 to 20 min. At predetermined intervals, the absorbance of the generated oxidized TMB was measured at 652.0 nm to evaluate the progression of the catalytic reaction and identify the minimum incubation period required to obtain a stable maximum signal. The reaction time that produced the optimal analytical response was selected for all subsequent experimental studies.

### 2.7. Colorimetric detection of hydrogen peroxide

The analytical capability of the Fe–Cu BNP nanozyme toward hydrogen peroxide was evaluated using a colorimetric assay under previously optimized reaction conditions. To identify the characteristic optical response of the catalytic system, the UV-Vis absorption spectrum generated from the Fe–Cu BNP-mediated oxidation of TMB was first collected over the wavelength range of 300.0–800.0 nm. Following spectral characterization, hydrogen peroxide solutions with progressively increasing concentrations were added to the reaction mixture while all remaining experimental variables were kept unchanged. The absorbance of the blue oxidized TMB product was then measured at 652.0 nm and the recorded signals were plotted against the corresponding H_2_O_2_ concentrations to establish the calibration relationship for quantitative hydrogen peroxide determination.

### 2.8. Arduino-integrated and smartphone-assisted colorimetric detection system

To enable portable and quantitative colorimetric measurements, the Fe–Cu BNP sensing platform was integrated with two complementary digital detection approaches comprising an Arduino-based RGB detection system and smartphone-assisted digital image analysis. Following completion of the colorimetric reaction under the optimized experimental conditions, the developed optical signal was independently evaluated using the two portable detection modes. Standardized illumination and sample positioning were maintained throughout the measurements to minimize variations arising from ambient lighting and geometrical differences. The Arduino- and smartphone-based sensing platforms were configured and applied as described in the following sections.

#### 2.8.1. Arduino-assisted colorimetric detection

The Arduino-assisted portable detection system consisted of a TCS34725 RGB color sensor interfaced with an Arduino Nano microcontroller through the I^2^C communication protocol. The TCS34725 sensor contains integrated photodiodes for detection of the red, green, blue, and clear components of incident light. The detected optical intensities were converted into digital signals using the integrated analog-to-digital converter of the sensor. The sensor integration time was set to 700 ms to provide stable acquisition of high-resolution 16-bit digital data.

Following completion of the Fe–Cu BNP-catalyzed TMB oxidation reaction, the reaction mixture was transferred into a clean cuvette and positioned at a fixed location within a light-shielded detection chamber to minimize interference from external illumination. The sample was illuminated under fixed conditions and the transmitted light was detected by the TCS34725 RGB sensor. The resulting digital signals were transferred through the I^2^C interface to the Arduino Nano microcontroller for acquisition and processing.

The Arduino-assisted system was programmed to sequentially acquire and process the optical response generated by the Fe–Cu BNP sensing platform. Following initialization of the Arduino Nano and TCS34725 RGB sensor, the blank/reference solution was measured to obtain the reference red-channel intensity *I*_0_. The reacted sample was subsequently measured under identical conditions to obtain the corresponding red-channel intensity (*I*), which was selected because of its high sensitivity toward the blue oxTMB product. The absorbance-equivalent response was calculated according to *A* = log_10_(*I*_0_/*I*) and subsequently converted to analyte concentration using the corresponding linear calibration equation. The programmed procedure therefore included reference and sample signal acquisition, red-channel processing, response calculation, calibration-based quantification, and output of the final analytical result.

Calibration curves were constructed by analyzing standard solutions of increasing analyte concentrations under identical experimental conditions. The processed Arduino response was plotted against the corresponding analyte concentration, and linear regression analysis was performed to obtain the calibration equation, slope, intercept, and coefficient of determination (*R*^2^). The resulting regression equation was subsequently employed for quantitative determination of the analyte in unknown samples. All measurements were performed in triplicate.

#### 2.8.2. Smartphone-assisted digital image analysis

For smartphone-assisted colorimetric analysis, the reaction mixture containing the developed blue oxidized TMB product was transferred into a clean cuvette and placed inside a custom-built light-shielded imaging chamber (30 × 30 × 28 cm) to minimize interference from ambient illumination. Uniform illumination was provided using a 6000 K white LED source and an iPhone 15 Pro smartphone (Apple Inc., USA) was positioned at a fixed distance of 24 cm from the sample. All images were acquired under identical illumination, sample positioning, and camera conditions to ensure reproducible digital color measurements. These imaging conditions are consistent with the portable smartphone-based configuration described in the related study.

The acquired images were imported into ImageJ software (National Institutes of Health, Maryland, USA) for quantitative digital image analysis. An identical region of interest (ROI) within the colored solution was selected for all standard and sample images to maintain consistent image-processing conditions. Each image was separated into its red (R), green (G), and blue (B) components and the mean intensity of each channel within the selected ROI was recorded. The red (R) channel was selected as the quantitative analytical signal because it provided the highest analytical sensitivity and strongest linear relationship with analyte concentration.

For calibration, images obtained from standard solutions containing increasing analyte concentrations were processed using the same ROI and image-analysis procedure. The mean red-channel intensity was plotted against the corresponding analyte concentration, and linear regression analysis was performed to obtain the calibration equation and coefficient of determination (*R*^2^). The resulting regression equation was subsequently used for quantitative determination of unknown samples. All image-based measurements were performed in triplicate.

### 2.9. Kinetic investigation of Fe–Cu BNPs

The steady-state kinetic behavior of the Fe–Cu BNPs was investigated using the catalytic oxidation of TMB in the presence of H_2_O_2_. Kinetic measurements were performed under the optimized catalytic conditions using 0.2 M acetate buffer (pH 4.0) at 40 °C. The TMB concentration was systematically varied while the concentrations of H_2_O_2_ and Fe–Cu BNPs and the other reaction conditions were maintained constant.

Immediately after initiation of the reaction, the formation of oxidized TMB (oxTMB) was monitored spectrophotometrically at 652.0 nm as a function of time. The initial reaction velocity (*ν*_0_) at each TMB concentration was determined from the slope of the initial linear portion of the corresponding absorbance–time profile. The obtained initial velocities were plotted against the corresponding TMB concentrations and analyzed according to the Michaelis–Menten kinetic model:
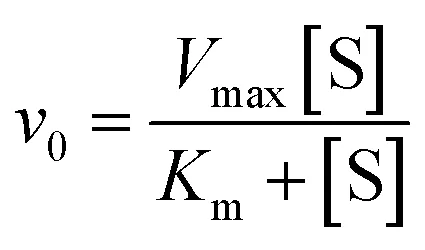
where *ν*_0_ is the initial reaction velocity, [S] is the TMB concentration, *V*_max_ is the maximum reaction velocity, and *K*_m_ is the Michaelis–Menten constant.

The kinetic parameters were additionally evaluated using the Lineweaver–Burk transformation:
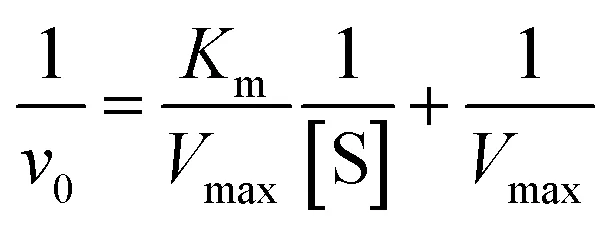


All kinetic measurements were performed under identical experimental conditions and the obtained kinetic parameters were used to evaluate the substrate affinity and catalytic efficiency of the Fe–Cu BNP nanozyme.

### 2.10. Glucose detection using the GOx/Fe–Cu BNP cascade system

A colorimetric assay for glucose determination was developed based on a cascade reaction involving glucose oxidase (GOx) and Fe–Cu BNP nanozymes. In the first step, 200 µL of glucose standard solutions covering the concentration range of 10–500 µM were mixed with 20 µL of GOx solution (40 IU mL^−1^) corresponding to 0.8 IU of GOx per assay in 50 mM acetate buffer (pH 5.1). The mixture was incubated at 37 °C for 30 min to allow GOx-catalyzed oxidation of glucose and the subsequent generation of H_2_O_2_.

Following the enzymatic reaction, 500 µL of Fe–Cu BNP suspension (1.0 mg mL^−1^) and 500 µL of TMB solution (5.0 mM) were added and the reaction mixture was adjusted with 0.2 M acetate buffer (pH 4.0). The mixture was subsequently incubated at 40 °C for 10 min. The H_2_O_2_ generated during glucose oxidation activated the peroxidase-like catalytic activity of the Fe–Cu BNPs promoting the oxidation of TMB to its characteristic blue-colored oxidized form. The absorbance was measured at 652.0 nm and the recorded absorbance values were plotted against the corresponding glucose concentrations to construct the calibration curve for quantitative glucose determination. The limits of detection (LOD) and quantification (LOQ) were calculated from the calibration data according to LOD = 3.3*σ*/*S* and LOQ = 10*σ*/*S*, where *σ* represents the standard deviation of replicate blank responses and *S* represents the slope of the corresponding calibration curve. The LOD and LOQ values were calculated independently for the spectrophotometric, Arduino-based, and smartphone-based detection modes.

### 2.11. Assessment of analytical selectivity toward glucose

Selectivity of the GOx/Fe–Cu BNP sensing platform was investigated using several potential interfering compounds commonly present in biological samples. Individual experiments were performed under the same optimized conditions employed for glucose determination using fructose, sucrose, lactose, ascorbic acid, and uric acid. The obtained colorimetric responses were compared with those produced by glucose at identical concentrations. The results demonstrated a significantly stronger response toward glucose while the tested interferents generated only negligible signals confirming the favorable selectivity of the developed GOx/Fe–Cu BNP sensing system. To further evaluate the response selectivity of the Fe–Cu BNP nanozyme independently of GOx, different oxidizing species capable of promoting TMB oxidation were investigated. Hydrogen peroxide (H_2_O_2_), *tert*-butyl hydroperoxide (TBHP), persulfate (S_2_O_8_^2−^), and hypochlorite (ClO^−^) were individually tested at an identical concentration of 100 µM under the optimized Fe–Cu BNP/TMB reaction conditions. Following incubation at 40 °C for 10 min in 0.2 M acetate buffer (pH 4.0), the absorbance of oxTMB was measured at 652.0 nm. All measurements were performed in triplicate.

### 2.12. Colorimetric detection of hexavalent chromium [Cr(vi)]

The colorimetric sensing performance of the Fe–Cu BNP platform toward hexavalent chromium [Cr(vi)] was investigated using potassium dichromate (K_2_Cr_2_O_7_) as the Cr(vi) source. Cr(vi) standard solutions covering the concentration range of 0–500 µM were prepared in double-distilled water. For each determination, 100 µL of the corresponding Cr(vi) standard solution was introduced into the Fe–Cu BNP/TMB reaction system containing 500 µL of Fe–Cu BNP suspension (1.0 mg mL^−1^) and 500 µL of TMB solution (5.0 mM). The reaction volume was adjusted with 0.2 M acetate buffer (pH 4.0) and the resulting mixture was incubated at 40 °C for 10 min.

Following incubation, the resulting color development was monitored by UV-Vis spectroscopy over the wavelength range of 300.0–800.0 nm and the absorbance of the oxidized TMB product was recorded at 652.0 nm. The recorded absorbance values were plotted against the corresponding Cr(vi) concentrations to establish the calibration curve for quantitative Cr(vi) determination.

To clarify the contribution of Fe–Cu BNPs to the Cr(vi) induced oxidation of TMB, control experiments were performed under identical experimental conditions using TMB alone, TMB with Fe–Cu BNPs, TMB with Cr(vi) in the absence of Fe–Cu BNPs, and the complete TMB/Cr(vi)/Fe–Cu BNP system. The UV-Vis spectra of the four systems were recorded and the absorbance of oxTMB at approximately 652.0 nm was compared to distinguishing the direct oxidation of TMB by Cr(vi) from the signal enhancement associated with the Fe–Cu BNPs.

### 2.13. Selectivity assessment for Cr(vi) detection

The selectivity of the Fe–Cu BNP-based Cr(vi) sensing system was evaluated using several metal ions commonly encountered in environmental water samples. Individual experiments were carried out under the same optimized conditions employed for Cr(vi) determination using Cr(iii), Fe(iii), Cu(ii), Zn(ii), Ni(ii), Co(ii), Pb(ii), Cd(ii), Hg(ii), and Mn(ii). The absorbance responses obtained for these ions were compared with that of Cr(vi) at identical concentrations. The selectivity of the proposed platform was assessed based on its ability to generate a significantly stronger response toward Cr(vi) than toward the tested interfering species.

### 2.14. Validation using biological samples

The practical applicability of the proposed GOx/Fe–Cu BNP sensing platform was evaluated through glucose determination in six independent human serum samples. Prior to analysis, each serum sample was diluted 20-fold with 0.2 M acetate buffer (pH 4.0) to bring the glucose concentration within the established linear working range of 10–500 µM. The glucose concentration in the original serum sample was calculated by multiplying the concentration obtained from the corresponding calibration curve by the dilution factor.

For comparative evaluation, glucose concentrations in the same serum samples were independently measured using a commercial glucose assay kit according to the manufacturer's recommended procedure. The results obtained using the commercial glucose meter were used as reference values for assessing the accuracy of the proposed GOx/Fe–Cu BNP sensing platform. All measurements using the proposed method were performed in triplicate and the results were expressed as mean ± standard deviation (SD). Agreement between the proposed and reference methods was statistically evaluated using a paired Student's *t*-test at the 95% confidence level (*α* = 0.05). The accuracy of the proposed method and the possible influence of the serum matrix were further investigated using standard-addition recovery experiments. Known concentrations of glucose were added to the serum samples before analysis and recovery was calculated as Recovery (%) = [(*C*_spiked_ − *C*_original_)/*C*_added_] × 100, where *C*_spiked_ and *C*_original_ represent the glucose concentrations determined in the spiked and original serum samples, respectively, and *C*_added_ represents the concentration of glucose added. Precision was evaluated as the relative standard deviation (RSD%) of replicate measurements.

### 2.15. Sustainability and analytical performance assessment

A comprehensive evaluation of the sustainability profile and analytical performance of the developed Fe–Cu BNP-based platform was performed using the Nanomaterial Assessment Tool (NAT), Environmental Performance-Practicality Index (EPPI), Blue Applicability Grade Index (BAGI), and Red Analytical Performance Index (RAPI). These assessment tools were applied according to their respective published protocols to evaluate different aspects of the proposed methodology including reagent safety, nanomaterial sustainability, energy consumption, waste generation, operational simplicity, practical applicability, and analytical performance. The individual criteria were evaluated according to the scoring framework of each assessment tool and the resulting scores were used to provide an integrated assessment of the environmental, practical, and analytical characteristics of the developed methodology.

## 3. Results and discussion

The eco-conscious synthesis strategy successfully produced Fe–Cu bimetallic nanoparticles with suitable structural characteristics and peroxidase-like catalytic activity. The interaction between iron and copper active centers enhanced the catalytic oxidation of TMB in the presence of hydrogen peroxide generating a blue-colored product suitable for quantitative colorimetric analysis. The developed nanozyme system exhibited favorable catalytic activity, storage stability, and substrate affinity enabling sensitive detection of glucose and Cr(vi). Furthermore, the integration of smartphone-assisted colorimetry and Arduino-based signal acquisition provided portable analytical approaches for quantitative measurements. The observed analytical performance and sustainability scores support the potential of the Fe–Cu nanozyme platform for biomedical sensing and future environmental applications.

### 3.1. Physicochemical investigation of Fe–Cu BNPs

The morphology, particle size, and crystallinity of the synthesized Fe–Cu BNPs were investigated using SEM, TEM, and SAED analyses. SEM was employed to examine the surface morphology and aggregation behavior, while TEM was used to determine the nanoscale dimensions of the particles and SAED was applied to assess their crystalline nature. SEM micrographs revealed the formation of densely packed nanoscale particles with irregular morphologies that aggregated into interconnected clusters generating a rough and porous surface architecture; Fig. 1. Such structural features are advantageous for catalytic applications because they increase surface accessibility and facilitate reactant diffusion. TEM images further confirmed the nanoscale dimensions of the synthesized material showing particle sizes ranging from 6.49 to 16.30 nm with an average diameter of approximately 11.7 nm; Fig. 2. The nanoparticles were assembled into network-like aggregates with overlapping layers providing a high surface area that may enhance catalytic efficiency. The selected-area electron diffraction (SAED) pattern displayed a series of well-defined concentric diffraction rings demonstrating the polycrystalline structure of the synthesized Fe–Cu BNPs and revealing the existence of multiple nanocrystalline domains within the particles; Fig. 3.

**Fig. 1:**
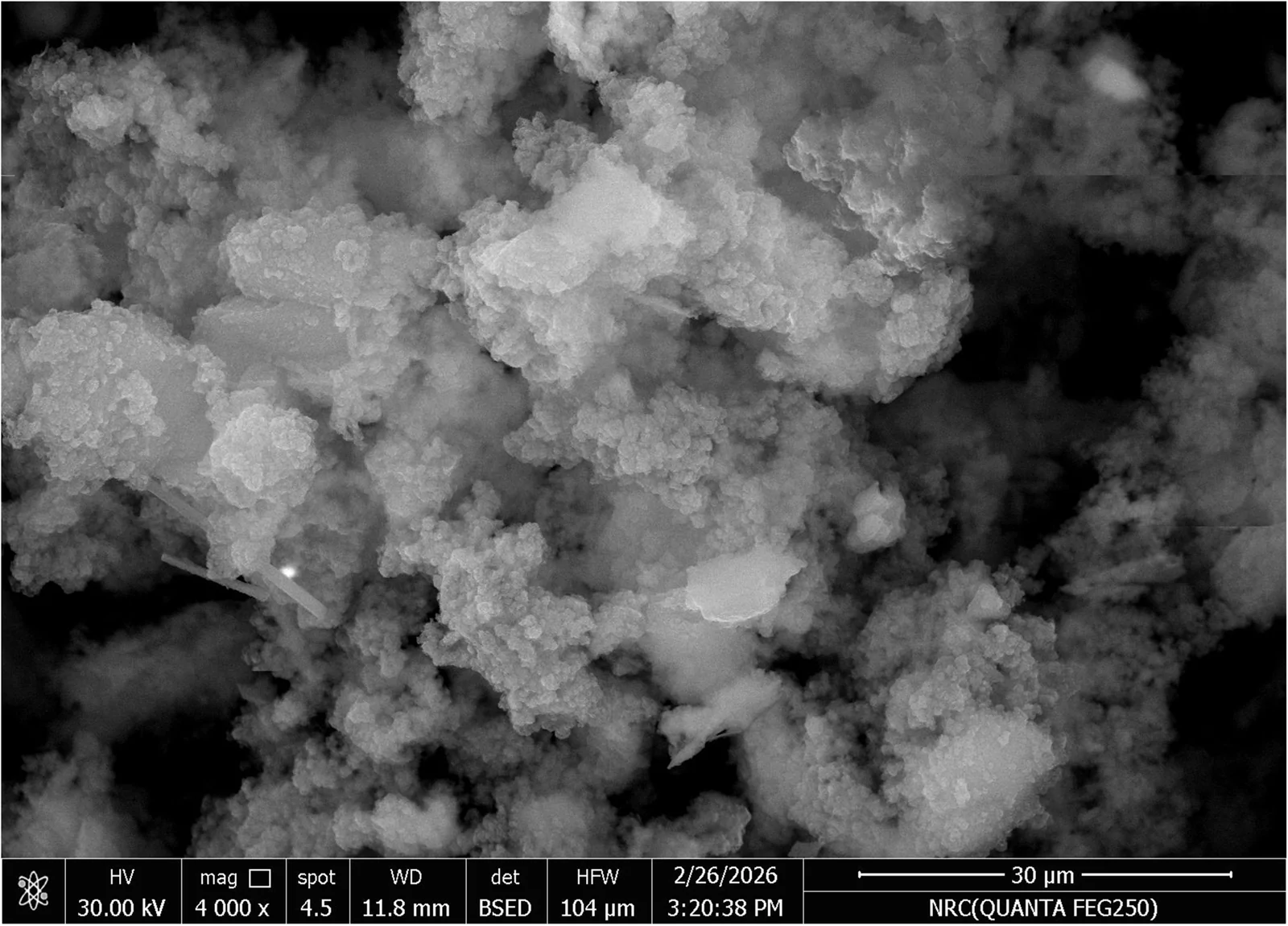
SEM micrograph of synthesized Fe–Cu BNPs at 4000× magnification. The image reveal agglomerated nanoparticulate structures with irregular morphology and porous architecture indicating the successful formation of nanoscale Fe–Cu assemblies.

**Fig. 2:**
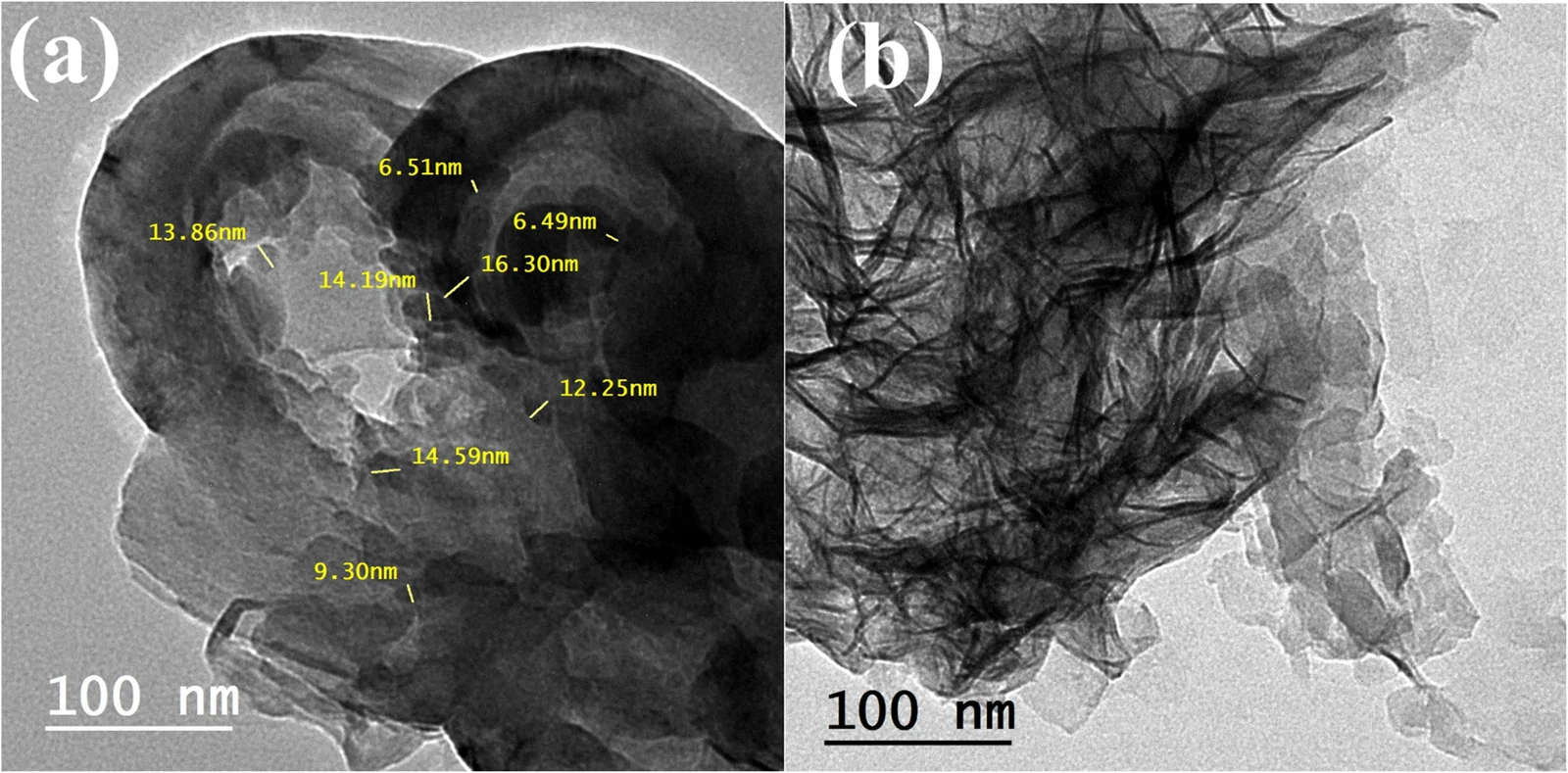
TEM images of synthesized Fe–Cu BNPs: (a) TEM image showing nanoscale particles with measured diameters ranging from approximately 6.5 to 16.3 nm and (b) TEM image illustrating the aggregated and layered morphology of the nanoparticle assemblies. The scale bar corresponds to 100 nm.

**Fig. 3:**
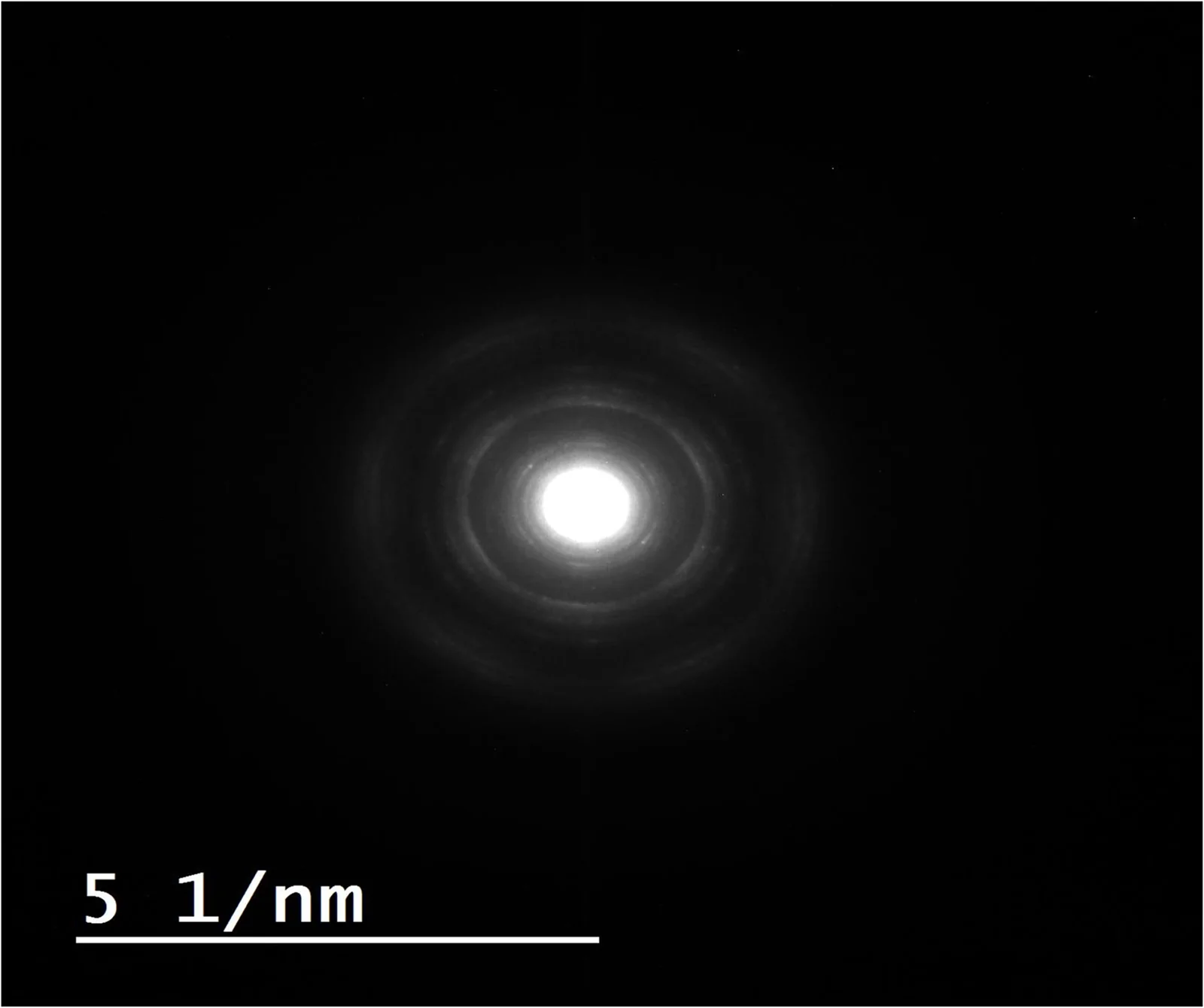
Selected Area Electron Diffraction (SAED) pattern of the synthesized Fe–Cu BNPs. The diffraction pattern exhibits distinct concentric rings corresponding to the polycrystalline nature of the nanoparticles. The diffuse and broadened rings indicate the nanocrystalline structure and small crystallite size of the synthesized Fe–Cu BNPs.

EDS analysis and elemental mapping were performed to verify the elemental composition of the synthesized Fe–Cu BNPs and to evaluate the spatial distribution of Fe and Cu within the nanostructures. EDS analysis revealed the presence of Fe, Cu, O, and C with weight percentages of 65.42%, 27.18%, 6.97%, and 0.43%, respectively; Fig. 4. Elemental mapping further demonstrated the homogeneous co-distribution of Fe and Cu throughout the examined nanostructures; Fig. 5. These findings confirm the presence and spatial association of both metallic components; however, EDS mapping alone cannot unequivocally establish the formation of a single bimetallic crystalline phase.

**Fig. 4:**
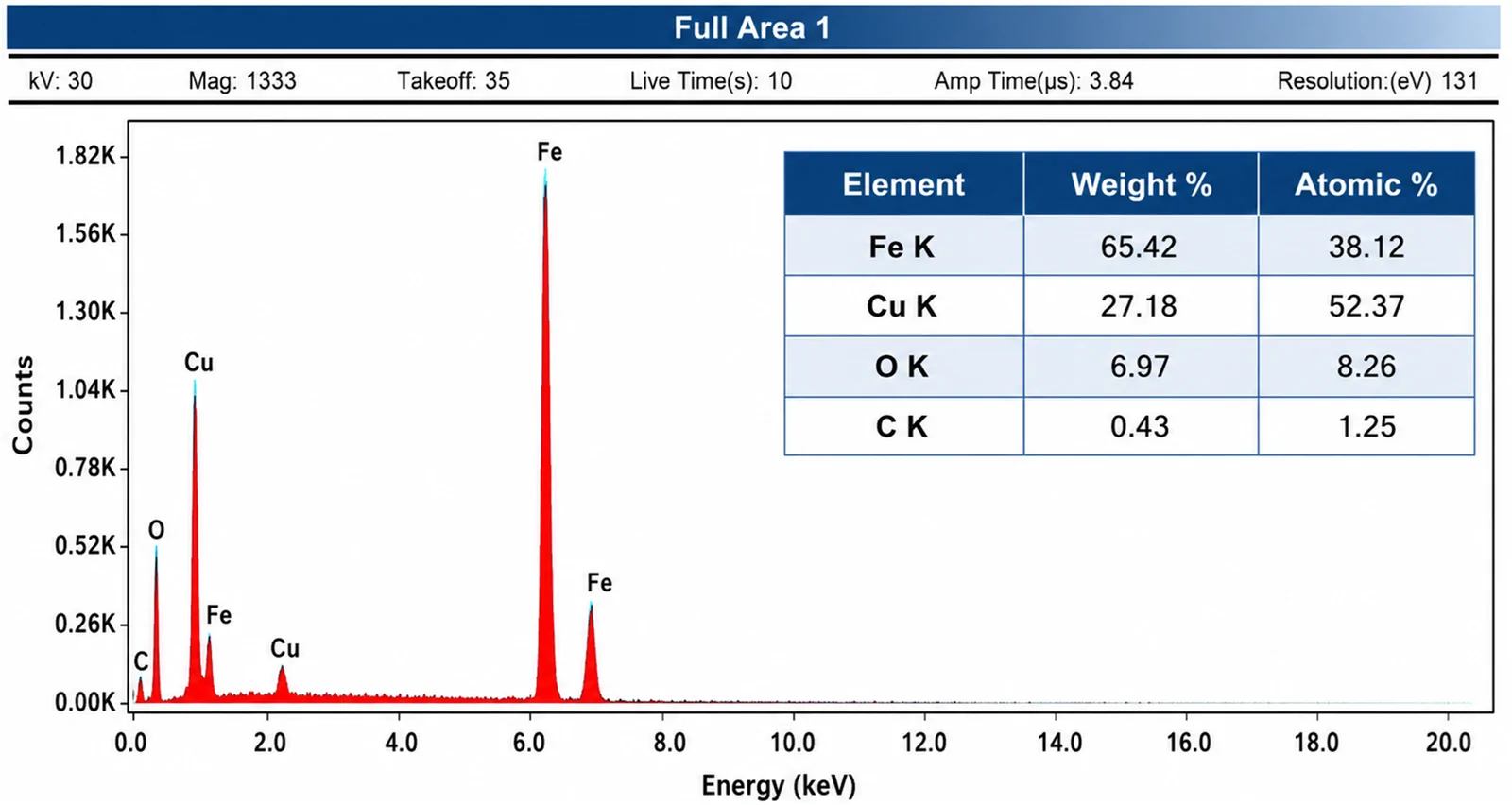
EDS spectrum of synthesized Fe–Cu BNPs confirms the presence of Fe, Cu, O, and C as the major elemental constituents.

**Fig. 5:**
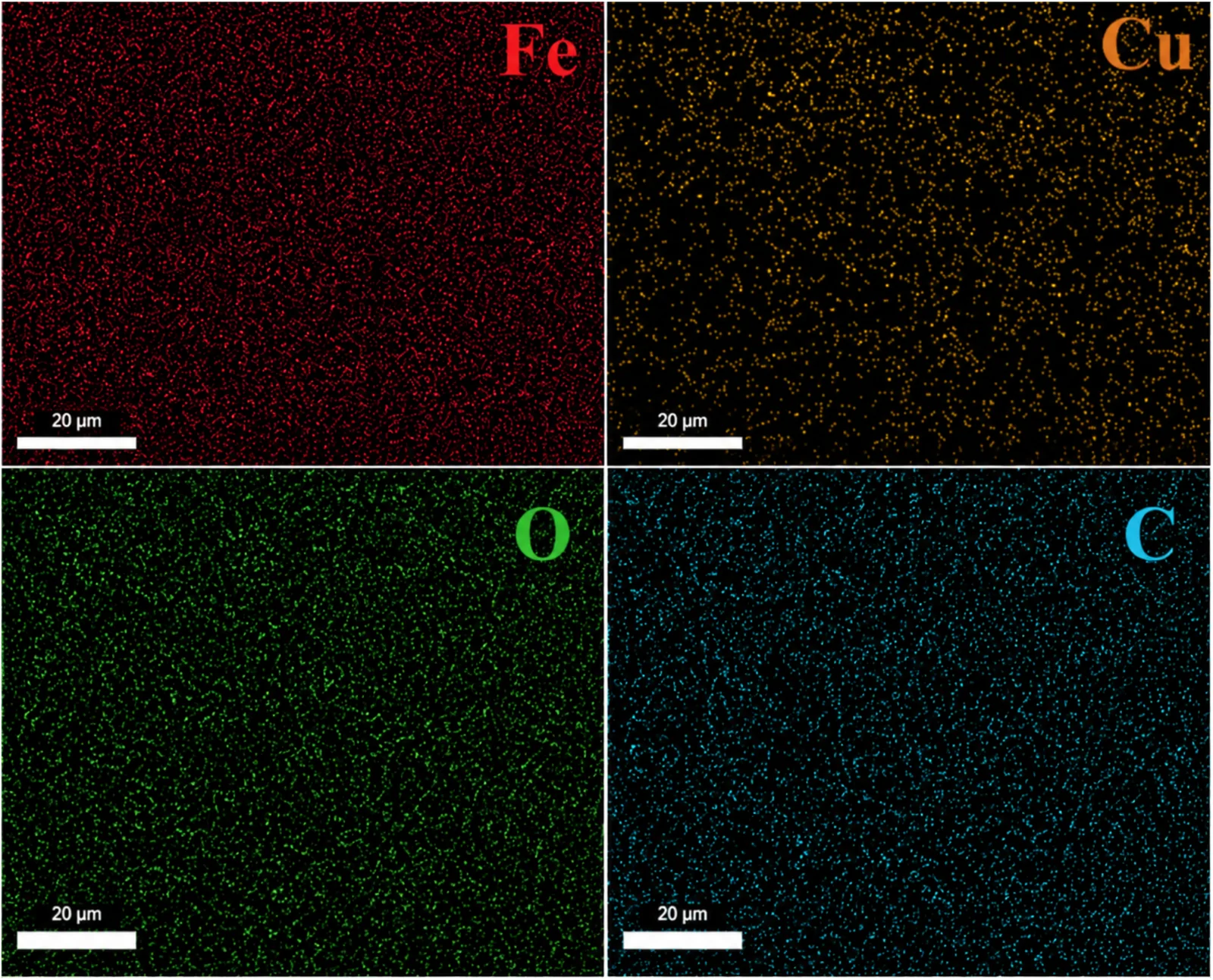
Elemental mapping images of synthesized Fe–Cu BNPs showing the spatial distribution of Fe, Cu, O, and C throughout the sample.

XRD analysis was performed to investigate the crystalline structure and identify the possible crystalline phases present in the synthesized Fe–Cu BNPs; Fig. S1. The prominent diffraction peaks observed at approximately 35°, 57°, and 63° (2*θ*) were consistent with characteristic reflections reported for tetragonal CuFe_2_O_4_. Specifically, these peaks were assigned to the (211), (321), and (224) crystallographic planes, respectively, in agreement with the standard CuFe_2_O_4_ reference pattern (JCPDS No. 34-0425) and previously reported diffraction data for CuFe_2_O_4_.^48^ The agreement between the experimental diffraction positions and the reference pattern supports the presence of a Cu–Fe spinel ferrite-type crystalline structure. Nevertheless, because related iron-oxide phases may exhibit overlapping diffraction reflections, the XRD and EDS results are considered supportive rather than unequivocal evidence for a single-phase CuFe_2_O_4_ structure. XPS analysis was performed to determine the surface chemical composition and oxidation states of Fe and Cu providing insight into the redox-active surface environment of the synthesized Fe–Cu BNPs; Fig. S2. Deconvolution of the Fe 2p spectrum revealed contributions from Fe^2+^ and Fe^3+^ species in the Fe 2p_3/2_ and Fe 2p_1/2_ regions accompanied by satellite features at approximately 718.5 and 731.2 eV indicating the coexistence of mixed iron oxidation states. The Cu 2p spectrum similarly exhibited contributions attributable to Cu^+^ and Cu^2+^ species together with characteristic satellite features at approximately 941 and 962 eV supporting the presence of Cu^2+^. The coexistence of multiple Fe and Cu oxidation states indicates a redox-active surface environment that may contribute to the electron-transfer processes underlying the observed peroxidase-like activity. Taken together, the XPS results, indexed XRD reflections, and homogeneous Fe/Cu distribution observed by EDS mapping provide complementary evidence for the incorporation of both Fe and Cu species within the synthesized nanostructures.

FTIR spectroscopy was employed to identify the surface functional groups and investigate metal–oxygen bonding and the possible presence of residual stabilizing species on the Fe–Cu BNP surface. FTIR analysis displayed a broad absorption band around 3400 cm^−1^ attributed to O–H stretching vibrations of surface hydroxyl groups and adsorbed water molecules while weak bands near 2900 cm^−1^ were associated with residual organic species; Fig. S3. Additional signals within the 1600–1000 cm^−1^ region corresponded to oxygen-containing surface functionalities whereas the absorption bands below 700 cm^−1^ were assigned to Fe–O and Cu–O vibrations supporting the presence of metal–oxygen bonding within the Fe–Cu-containing nanostructures. Collectively, the SEM, TEM, SAED, EDS mapping, XRD, XPS, and FTIR results support the successful formation of crystalline Fe–Cu-containing nanostructures with nanoscale dimensions, homogeneous elemental distribution, mixed Fe and Cu oxidation states, and a porous architecture, all of which are favorable characteristics for catalytic nanozyme applications.

### 3.2. Assessment of the catalytic performance of Fe–Cu BNPs

The catalytic capability of the synthesized Fe–Cu BNPs was explored using a TMB/H_2_O_2_ reaction system where color development served as an indicator of nanozyme activity; Fig. 6. Control experiments containing individual components or incomplete reaction mixtures exhibited negligible color formation indicating limited TMB oxidation under these conditions. Conversely, the complete reaction system comprising Fe–Cu BNPs, TMB, and H_2_O_2_ rapidly generated an intense blue color together with a prominent absorption peak at 652.0 nm confirming the efficient oxidation of TMB.

**Fig. 6:**
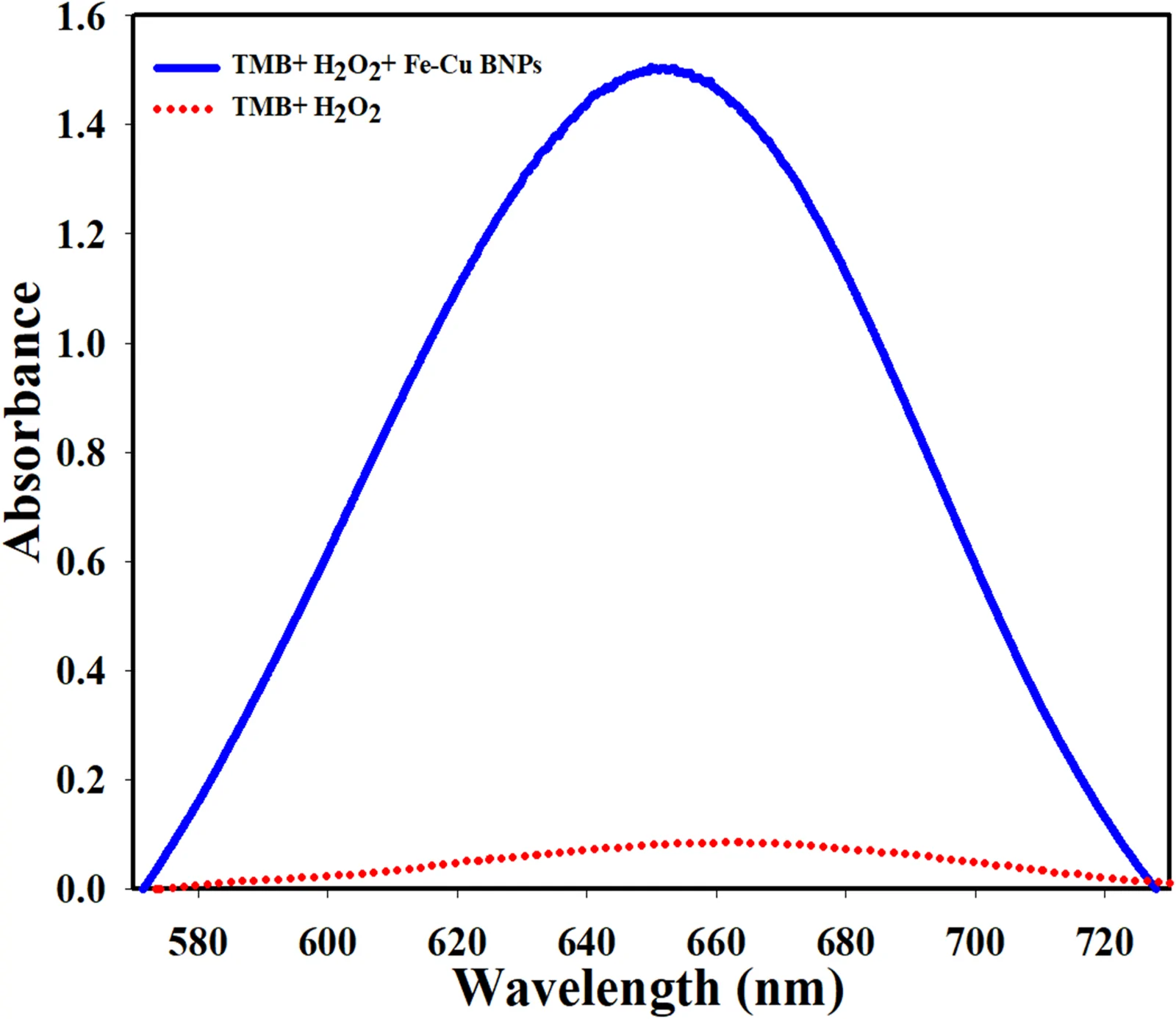
UV-Vis absorption spectra demonstrating the peroxidase-like catalytic activity of Fe–Cu BNPs. The TMB/H_2_O_2_/Fe–Cu BNP system exhibited a pronounced absorption peak at approximately 652.0 nm corresponding to the formation of oxidized TMB (oxTMB), whereas the TMB/H_2_O_2_ system in the absence of Fe–Cu BNPs showed only a weak absorbance response.

The cooperative contribution of Fe and Cu to the observed peroxidase-like activity was further investigated through comparative catalytic control experiments. As shown in Fig. S4, the Fe–Cu BNPs exhibited a markedly higher oxTMB absorbance at 652.0 nm than the Fe-only NPs, Cu-only NPs, and the corresponding physical Fe/Cu mixture under identical reaction conditions. Notably, the enhanced catalytic response of the Fe–Cu BNPs relative to the physical mixture supports a beneficial cooperative interaction between Fe and Cu within the synthesized nanostructure rather than a simple additive contribution of the individual components.

The involvement of reactive oxygen species in the catalytic process was further investigated by EPR spin-trapping analysis using DMPO. As shown in Fig. S5, radical-adduct signals assigned to hydroxyl- and superoxide-derived species were observed for the Fe–Cu BNP/H_2_O_2_ catalytic system, whereas the DMPO blank showed no appreciable EPR signal. These observations support ROS generation during the Fe–Cu BNP-mediated peroxidase-like reaction. Furthermore, the coexistence of Fe^2+^/Fe^3+^ and Cu^+^/Cu^2+^ surface species identified by XPS indicates a redox-active surface environment that may facilitate electron-transfer processes. Collectively, the comparative catalytic controls, EPR results, and XPS analysis provide complementary experimental support for the proposed cooperative Fe–Cu redox activity and ROS-mediated oxidation of TMB.

### 3.3. Evaluation of parameters affecting catalytic efficiency

#### 3.3.1. Influence of pH on the oxidative catalytic response

The influence of pH on the catalytic activity of the Fe–Cu BNPs was investigated over the pH range of 2.0–8.0 using appropriate buffer systems for the different pH regions as described in Section 2.6.1; the results are presented in Fig. 7a. The catalytic response exhibited a pronounced dependence on the acidity of the reaction medium. The absorbance increased markedly as the pH approached 4.0, reaching its highest value under these conditions. In contrast, both strongly acidic and near-neutral media produced significantly lower signals. The superior performance at pH 4.0 can be attributed to more effective activation of hydrogen peroxide and improved electron-transfer interactions between the Fe–Cu active sites and the TMB substrate. Therefore, pH 4.0 was selected as the optimum condition for all subsequent experiments.

**Fig. 7:**
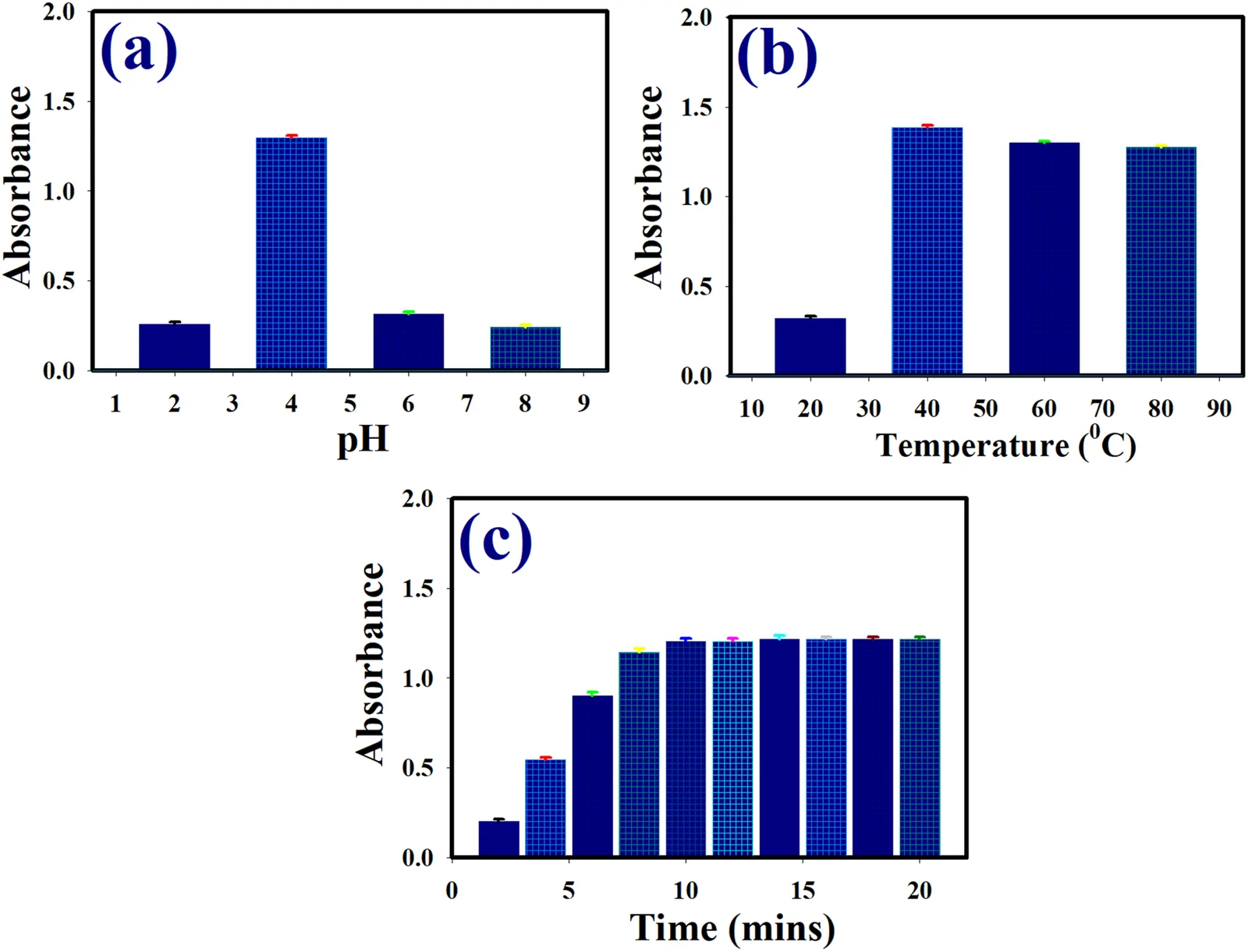
Optimization of the catalytic reaction conditions for Fe–Cu BNPs-mediated TMB oxidation. Effect of (a) pH, (b) reaction temperature, and (c) incubation time on the absorbance intensity of oxidized TMB. Experimental conditions were systematically varied to identify the optimum parameters yielding the highest catalytic response. Error bars represent the standard deviation of three independent experimental determinations (*n* = 3).

#### 3.3.2. Effect of reaction temperature on catalytic activity

The influence of reaction temperature on the peroxidase-like oxidation of TMB catalyzed by the Fe–Cu nanozyme was systematically investigated over a temperature range of 20–80 °C; Fig. 7b. Increasing the temperature from 20 to 40 °C resulted in a substantial enhancement of absorbance indicating accelerated catalytic kinetics and more efficient substrate conversion. The maximum catalytic response was achieved at 40 °C. Further elevation of temperature caused a slight decrease in absorbance suggesting partial loss of catalytic efficiency due to reduced stability of reactive oxygen species or alterations in the surface catalytic environment. Accordingly, 40 °C was adopted as the optimum reaction temperature. Notably, this temperature is close to physiological conditions highlighting the suitability of the Fe–Cu BNP nanozyme system for potential bioanalytical and point-of-care diagnostic applications involving biological samples.

#### 3.3.3. Effect of reaction time

The influence of incubation time on the catalytic oxidation process was examined over the range of 2–20 min as shown in Fig. 7c. The absorbance increased steadily during the initial stages of the reaction reflecting continuous formation of the oxidized TMB product. A rapid increase was observed up to approximately 10 min after which the signal reached a nearly constant value with only negligible changes at longer incubation periods. This plateau behavior indicates that the catalytic reaction had approached completion. Based on these findings, an incubation period of 10 min was adopted for all subsequent experiments as it provided a high analytical response while preserving the rapidity of the assay.

Overall, the optimum catalytic conditions for the Fe–Cu BNP-based sensing platform were established as pH 4.0 a reaction temperature of 40 °C, and an incubation time of 10 min. These conditions were employed throughout the subsequent analytical investigations to achieve maximum catalytic efficiency and measurement reproducibility.

### 3.4. Colorimetric detection of hydrogen peroxide

The sensing capability of the Fe–Cu BNP-based nanozyme system for hydrogen peroxide was investigated after establishing the most favorable operating conditions. As shown in Fig. 8a, the absorbance intensity at 652.0 nm increased progressively with increasing H_2_O_2_ concentration indicating enhanced oxidation of TMB catalyzed by the Fe–Cu active centers. The generated, blue-colored oxidized TMB species exhibited a concentration-dependent response over the investigated range confirming the efficient peroxidase-like activity of the synthesized nanozyme.

**Fig. 8:**
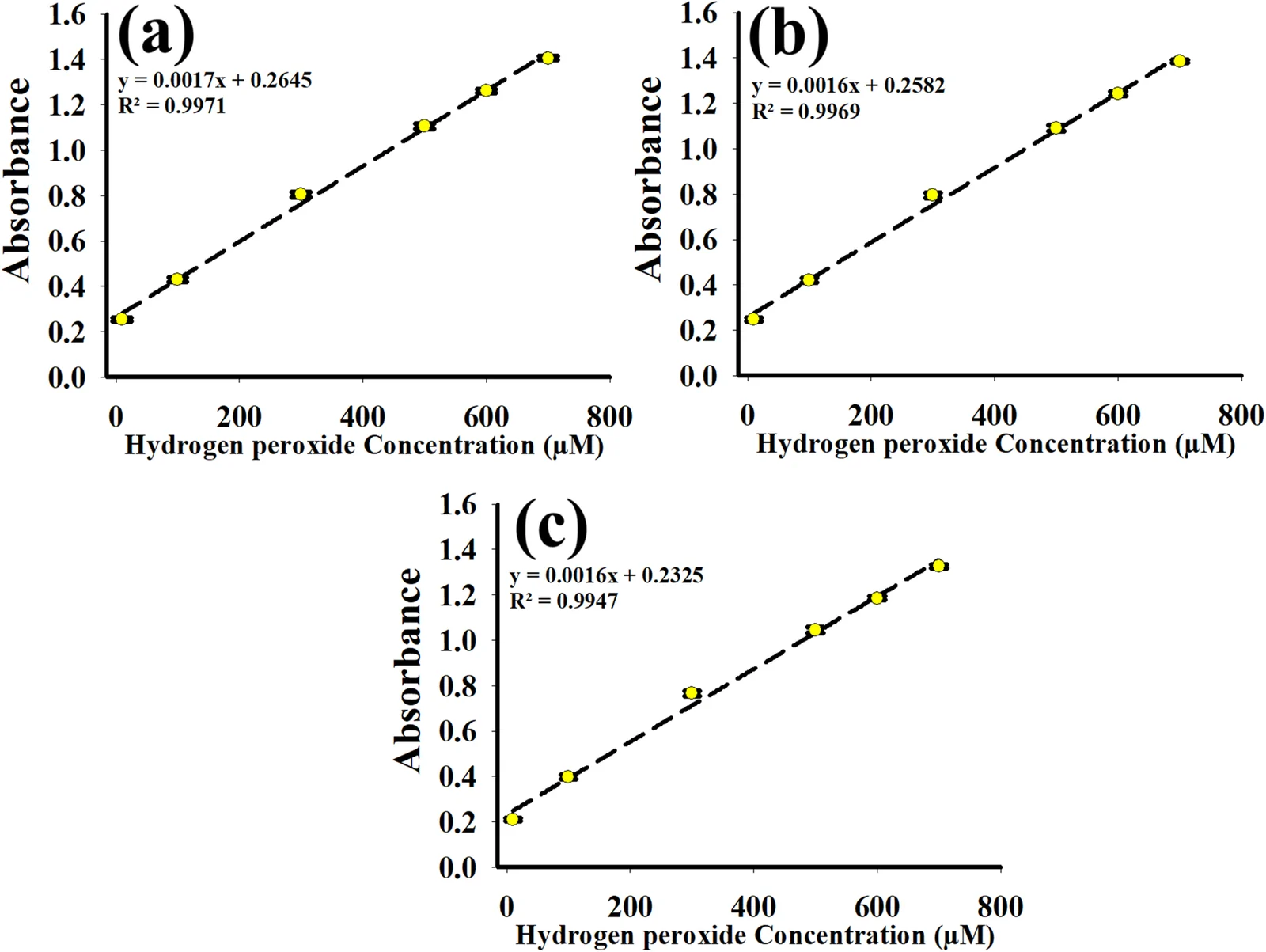
Calibration curves for hydrogen peroxide determination using Fe–Cu BNPs under optimized experimental conditions. (a) UV-Vis spectrophotometric detection at 652.0 nm, (b) Arduino-assisted signal acquisition, and (c) smartphone-based RGB image analysis. The corresponding regression equations and coefficients of determination demonstrate linear responses over the investigated hydrogen peroxide concentration range. Error bars represent the standard deviation of three independent experimental determinations (*n* = 3).

A linear relationship was observed over the investigated H_2_O_2_ concentration range, yielding the calibration equation *y* = 0.0017*x* + 0.2645 with a coefficient of determination (*R*^2^) of 0.9971 indicating good agreement between the experimental data and the regression model; Table 1. The obtained linearity demonstrates the capability of the Fe–Cu BNPs for quantitative determination of hydrogen peroxide. The observed sensitivity can be attributed to the synergistic interaction between iron and copper catalytic sites which promotes electron transfer and generation of reactive oxygen species during the catalytic process.

**Table 1:** Comparative analytical performance of spectrophotometric, Arduino-based, and smartphone-based methods for hydrogen peroxide detection using the fabricated Fe–Cu BNPs-based glucose sensing system

Parameter	Spectrophotometric method	Arduino-based system	Smartphone-based system
Linear range (µM)	10–700	10–700	10–700
Slope	0.0017	0.0016	0.0016
Intercept	0.2645	0.2582	0.2325
Correlation coefficient (*R*^2^)	0.9971	0.9969	0.9947
LOD (µM)	9.7	10.3	10.3
LOQ (µM)	29.4	31.3	31.3
Optimal pH	4.0	4.0	4.0
Incubation temperature (°C)	40	40	40
Reaction time (min)	10	10	10

### 3.5. Arduino-assisted and smartphone-based colorimetric analysis

To further enhance the on-site applicability of the proposed sensing system, the Fe–Cu BNP nanozyme system was coupled with Arduino-assisted signal acquisition and smartphone-based image analysis for instrument-independent hydrogen peroxide determination. The color change generated during the Fe–Cu BNP-catalyzed oxidation of TMB was successfully monitored using both portable detection approaches.

As shown in Fig. 8b, the Arduino-assisted platform exhibited a strong concentration-dependent response toward H_2_O_2_ over the investigated range. A highly linear calibration relationship was obtained with the regression equation *y* = 0.0016*x* + 0.2582 and a correlation coefficient of *R*^2^ = 0.9969; Table 1. The obtained linearity demonstrates the capability of the Arduino module to accurately quantify catalytic color development while simultaneously providing rapid digital signal acquisition and processing.

The applicability of smartphone-based detection was further evaluated through RGB image analysis of the developed colorimetric system. As illustrated in Fig. 8c, the smartphone-derived signals increased proportionally with increasing H_2_O_2_ concentration yielding the regression equation *y* = 0.0016*x* + 0.2325 with a correlation coefficient of *R*^2^ = 0.9947; Table 1. The high degree of agreement between smartphone measurements and spectrophotometric data confirms the reliability of image-based analysis for quantitative determination. Representative images used for smartphone-assisted colorimetric analysis and their subsequent processing using ImageJ are presented in Fig. S6. The iPhone-acquired images showed a progressive variation in the developed blue color across the investigated samples. For quantitative image analysis, an identical region of interest (ROI) within the liquid phase was applied to each sample followed by separation into the red, green, and blue channels using ImageJ. The red channel was selected for quantitative analysis because it provided the most sensitive response to changes in the blue oxTMB color intensity. These results demonstrate the applicability of smartphone imaging combined with ImageJ processing for quantitative colorimetric signal evaluation.

Comparison of the three detection approaches showed consistent analytical responses with coefficients of determination exceeding 0.99 in all cases. The results obtained confirm that the Fe–Cu BNP-based sensing system is capable of achieve reliable hydrogen peroxide quantification using either conventional spectrophotometry or portable digital devices. The successful integration of Arduino electronics and smartphone imaging significantly enhances the practicality of the proposed system for point-of-care diagnostics, field monitoring, and resource-limited analytical applications.

### 3.6. Kinetic investigation of Fe–Cu BNPs

The peroxidase-like catalytic behavior of the synthesized Fe–Cu BNPs was further investigated by steady-state kinetic analysis using TMB as the substrate. As the TMB concentration increased, the initial reaction velocity (*ν*_0_) progressively increased and approached a saturation region displaying characteristic Michaelis–Menten behavior. The relationship between the initial reaction velocity and TMB concentration was evaluated using the Michaelis–Menten model and the kinetic parameters were additionally assessed using the corresponding Lineweaver–Burk transformation. The resulting Michaelis–Menten and Lineweaver–Burk plots are presented in Fig. S7.

The kinetic analysis yielded a Michaelis–Menten constant *K*_m_ of 0.18 mM and a maximum reaction velocity *V*_max_ of 8.92 × 10^−8^ M s^−1^. The relatively low *K*_m_ value indicates favorable interaction between TMB and the catalytic active sites of the Fe–Cu BNPs suggesting good substrate affinity. The obtained *V*_max_ reflects effective catalytic conversion of TMB under the investigated conditions. This catalytic behavior may be attributed to the cooperative redox activity of Fe and Cu centers which facilitates electron-transfer processes and promotes TMB oxidation in the presence of H_2_O_2_.

To further assess the catalytic efficiency of the developed nanozyme, its kinetic performance was compared with several previously reported peroxidase-mimicking nanomaterials^49–51^ as summarized in Table 2. The Fe–Cu BNPs exhibited a relatively low *K*_m_ value compared with the selected reported nanozyme systems indicating favorable substrate affinity. These findings demonstrate the potential of the synthesized Fe–Cu BNPs as an efficient nanozyme platform for sensitive colorimetric sensing applications.

**Table 2:** Comparison of kinetic parameters of Fe–Cu BNPs with selected reported nanozymes using TMB as substrate

Nanozyme system	*K*_m_ (mM)	*V*_max_	Reference
Fe_3_O_4_ nanoparticles	0.30	3.20 × 10^−8^ M s^−1^	49
CeO_2_ nanoparticles	0.21	5.60 × 10^−8^ M s^−1^	50
Fe_3_O_4_@CeO_2_ nanocomposites	0.16	7.90 × 10^−8^ M s^−1^	51
Fe–Cu BNPs	0.18	8.92 × 10^−8^ M s^−1^	This work

### 3.7. Glucose detection using the GOx/Fe–Cu BNP cascade system

Glucose quantification was achieved using a sequential catalytic approach in which glucose oxidase (GOx) generated hydrogen peroxide that was subsequently utilized by Fe–Cu BNPs exhibiting intrinsic peroxidase-mimicking activity to produce the colorimetric response. Initially, GOx catalyzed the oxidation of glucose to gluconic acid simultaneously generating hydrogen peroxide as a reaction by-product. The H_2_O_2_ generated during the reaction was subsequently utilized by Fe–Cu BNPs to catalyze the oxidation of TMB producing a blue oxidized species that exhibited a distinct absorbance peak at 652.0 nm.

As illustrated in Fig. 9a, a gradual increase in absorbance was observed as the glucose concentration increased, demonstrating the efficient coupling between GOx-generated H_2_O_2_ and the Fe–Cu BNP catalytic system. A highly linear relationship was obtained over the concentration range of 10–500 µM glucose with the regression equation *y* = 0.0017*x* + 0.1864 and an excellent correlation coefficient (*R*^2^ = 0.9981) confirming the high analytical sensitivity of the developed assay; Table 3.

**Fig. 9:**
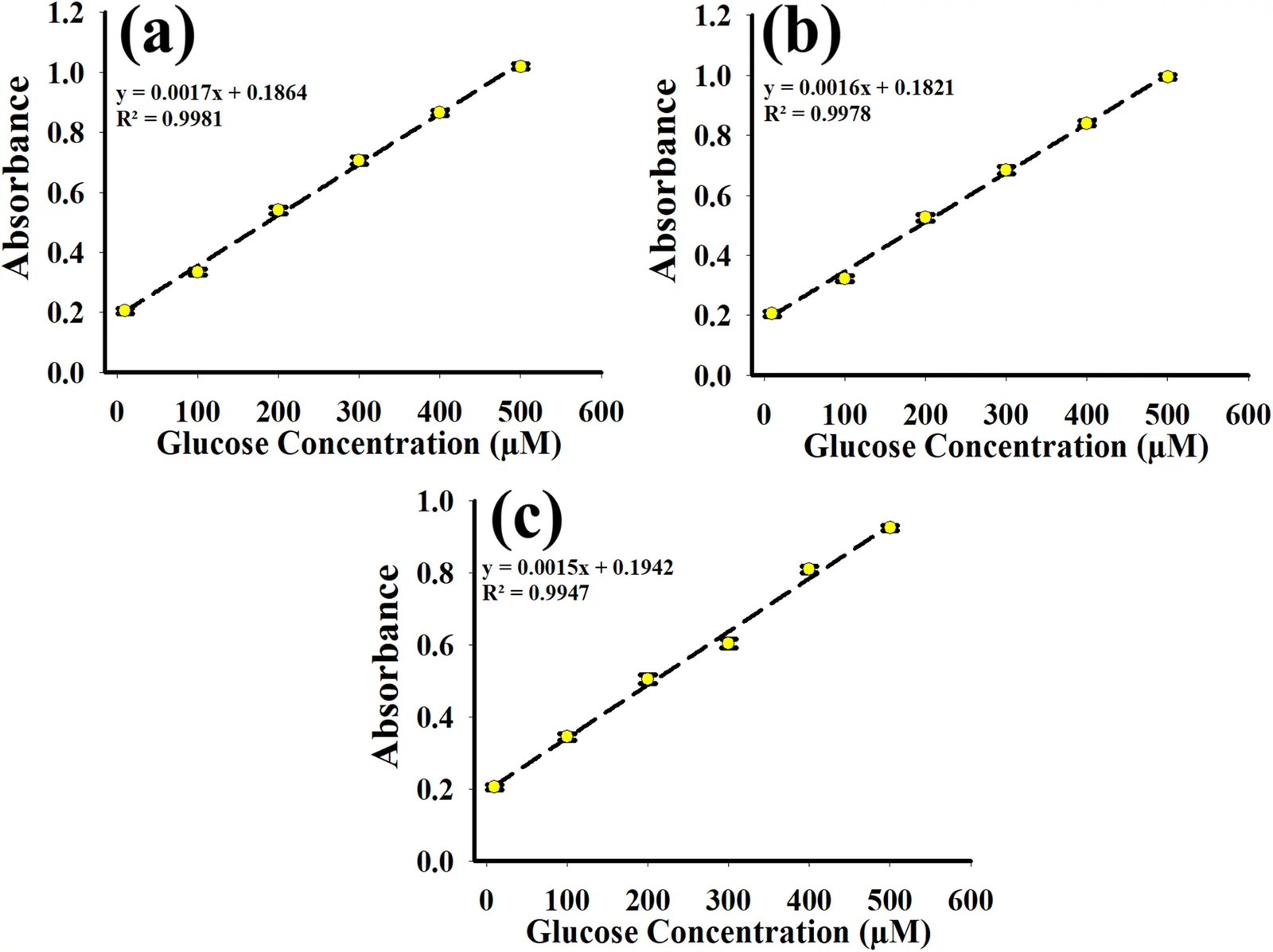
Calibration plots for glucose determination using the GOx/Fe–Cu BNP cascade sensing platform under optimized conditions. (a) UV-Vis spectrophotometric detection at 652.0 nm, (b) Arduino-assisted signal acquisition, and (c) smartphone-based RGB image analysis. The progressive increase in signal intensity with glucose concentration demonstrates the efficient generation of hydrogen peroxide by GOx and its subsequent utilization in Fe–Cu BNP-catalyzed TMB oxidation. The corresponding regression equations and coefficients of determination confirm linear responses over the investigated concentration range. Error bars represent the standard deviation of three independent experimental determinations (*n* = 3).

**Table 3:** Comparative analytical performance of spectrophotometric, Arduino-based, and smartphone-based methods for glucose detection using the GOx/Fe–Cu BNP cascade sensing system

Parameter	Spectrophotometric method	Arduino-based system	Smartphone-based system
Linear range (µM)	10–500	10–500	10–500
Slope	0.0017	0.0016	0.0015
Intercept	0.1864	0.1821	0.1942
Correlation coefficient (*R*^2^)	0.9981	0.9978	0.9947
LOD (µM)	9.7	10.3	11.0
LOQ (µM)	29.4	31.3	33.3
Optimal pH	4.0	4.0	4.0
Incubation temperature (°C)	40	40	40
Reaction time (min)	10	10	10

To extend the applicability of the sensing platform for portable analysis, an Arduino-assisted detection system was employed. As illustrated in Fig. 9b, the Arduino-derived signals exhibited a concentration-dependent increase throughout the investigated glucose range producing a linear calibration equation of *y* = 0.0016*x* + 0.1821 with a correlation coefficient of *R*^2^ = 0.9978; Table 3. The agreement with spectrophotometric measurements demonstrates the capability of the Arduino platform for reliable digital signal acquisition and quantitative glucose determination.

Furthermore, smartphone-based image analysis was performed by capturing the color changes generated during the catalytic reaction under controlled illumination conditions. As presented in Fig. 9c, the smartphone-derived response also increased proportionally with glucose concentration yielding a regression equation of *y* = 0.0015*x* + 0.1942 and a correlation coefficient of *R*^2^ = 0.9947; Table 3. The strong correlation obtained confirms the suitability of smartphone imaging as a convenient and an economical substitute for traditional analytical equipment.

Comparable analytical outcomes generated by laboratory spectroscopic measurements, smartphone-assisted color evaluation, and Arduino-based signal acquisition verify the consistency of the GOx/Fe–Cu BNP detection platform. The integration of accurate quantification, reliable concentration-dependent response, and user-friendly multimodal operation underscores its promise for near-patient glucose assessment and analytical testing beyond conventional laboratory settings.

### 3.8. Selectivity evaluation for glucose determination

The selectivity of the GOx/Fe–Cu BNP sensing platform was evaluated using common biological interferents namely fructose, lactose, sucrose, uric acid, and ascorbic acid were individually evaluated at a concentration of 200 µM using the same experimental protocol. As presented in Table 4, glucose produced a markedly stronger analytical response than any of the examined interferents whereas the remaining compounds induced only minimal variations in signal intensity. The observed selectivity can be explained by the inherent preference of glucose oxidase for glucose molecules and the effective catalytic signal generation mediated by Fe–Cu BNP. These results confirm the suitability of the developed platform for selective glucose determination in complex biological samples. To distinguish the substrate specificity of GOx from the response characteristics of the Fe–Cu BNP nanozyme, the catalytic response toward different oxidizing species was further investigated. As shown in Fig. S8, H_2_O_2_ exhibited the highest absorbance response (0.82), whereas substantially lower responses were observed for TBHP (0.28), S_2_O_8_^2−^ (0.24), and ClO^−^ (0.19) under identical experimental conditions. These results indicate that the Fe–Cu BNP/TMB system exhibited a preferential response toward H_2_O_2_ compared with the other investigated oxidizing species. Thus, while the selectivity toward glucose is primarily governed by the substrate specificity of GOx, the oxidant comparison demonstrates the response characteristics of the Fe–Cu BNP nanozyme toward different oxidizing species.

**Table 4:** Selectivity evaluation of the GOx/Fe–Cu BNP cascade sensing system toward glucose in the presence of common biological interferents

Interferent	Concentration (µM)	Relative response (%)
Glucose	200	98.0
Ascorbic acid	200	8.5
Uric acid	200	5.2
Fructose	200	7.9
Lactose	200	8.3
Sucrose	200	6.3
Citric acid	200	5.4

### 3.9. Colorimetric detection of Cr(vi)

The colorimetric sensing capability of the synthesized Fe–Cu BNPs toward hexavalent chromium [Cr(vi)] was investigated using the TMB-based sensing system. As shown in Fig. 10a, increasing Cr(vi) concentrations resulted in a progressive enhancement of the characteristic absorption band of oxTMB centered at approximately 652.0 nm accompanied by a corresponding increase in the intensity of the developed blue color. This concentration-dependent spectral response demonstrates the capability of the proposed sensing system to provide a readily measurable optical signal for Cr(vi) determination.

**Fig. 10:**
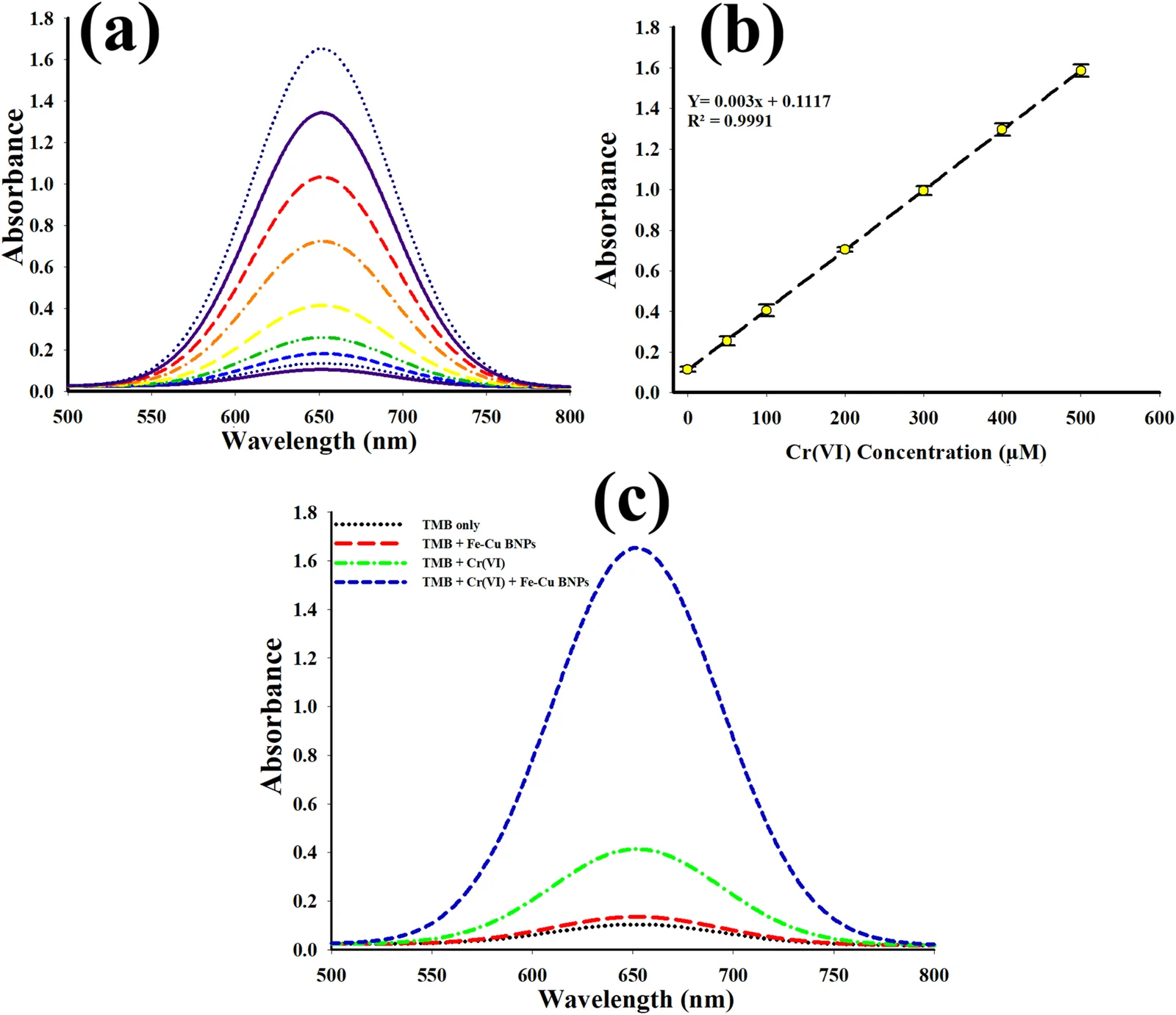
Colorimetric determination of Cr(vi) using the Fe–Cu BNP nanozyme platform. (a) UV-Vis absorption spectra recorded in the presence of increasing Cr(vi) concentrations (0–500 µM) showing a concentration-dependent enhancement of the characteristic oxTMB absorption band centered at approximately 652.0 nm. (b) Corresponding calibration curve constructed from the absorbance values measured at 652.0 nm showing a linear relationship over the investigated concentration range with the regression equation *y* = 0.003*x* + 0.1117 and *R*^2^ = 0.9991; error bars represent the standard deviation of three independent experimental determinations (*n* = 3). (c) UV-Vis spectra of the control systems comprising TMB alone, TMB + Fe–Cu BNPs, TMB + Cr(vi) in the absence of Fe–Cu BNPs, and the complete TMB + Cr(vi) + Fe–Cu BNP system demonstrating the contribution of Fe–Cu BNPs to enhancement of the Cr(vi)-induced colorimetric response.

The absorbance measured at 652.0 nm increased systematically with increasing Cr(vi) concentration over the investigated range of 0–500 µM. As illustrated in Fig. 10b, a highly linear calibration relationship was obtained according to the regression equation *y* = 0.003*x* + 0.1117 with a coefficient of determination of (*R*^2^ = 0.9991). The strong linear relationship demonstrates the analytical capability of the developed platform for quantitative Cr(vi) detection over the investigated concentration range.

To clarify the contribution of Fe–Cu BNPs to the Cr(vi) induced oxidation of TMB, control experiments were conducted under identical experimental conditions; Fig. 10c. TMB alone produced only a weak background response, while the TMB/Cr(vi) system exhibited a distinct oxTMB absorption band even in the absence of Fe–Cu BNPs. This observation confirms that Cr(vi) can directly oxidize TMB under the acidic reaction conditions. Importantly, the complete TMB/Cr(vi)/Fe–Cu BNP system exhibited a substantially higher absorbance than the TMB/Cr(vi) system without the nanozyme demonstrating that the presence of Fe–Cu BNPs markedly enhances the resulting colorimetric response.

Accordingly, the Cr(vi)-dependent response of the developed sensing platform is attributed to the combined contribution of direct TMB oxidation by Cr(vi) and signal enhancement in the presence of Fe–Cu BNPs. Therefore, the Fe–Cu BNPs are more appropriately considered a signal-amplifying component of the Cr(vi) sensing system rather than the sole origin of TMB oxidation. The concentration-dependent response, good linearity, and enhanced optical signal obtained in the presence of Fe–Cu BNPs demonstrate the promising analytical performance of the proposed platform for Cr(vi) detection and indicate its potential for future environmental monitoring applications. However, further validation using real environmental samples is required to establish its analytical performance in complex environmental matrices.

The proposed mechanism underlying the Cr(vi) colorimetric response is illustrated in Fig. S9. Under the acidic assay conditions, Cr(vi) species act as strong oxidizing agents and electron acceptors promoting the oxidation of colorless TMB to blue oxTMB. The Fe–Cu BNPs provide cooperative Fe^3+^/Fe^2+^ and Cu^2+^/Cu^+^ redox-active sites that may facilitate interfacial electron-transfer processes and enhance the resulting colorimetric response. Consequently, increasing Cr(vi) concentration promotes oxTMB formation producing a concentration-dependent increase in absorbance at 652.0 nm. This proposed mechanism is consistent with the control experiments and the mixed Fe and Cu oxidation states identified by XPS supporting the role of the Fe–Cu BNPs as a redox-active interface in Cr(vi)-mediated TMB oxidation (Fig. S9).

### 3.10. Selectivity evaluation for Cr(vi) detection

The selectivity of the Fe–Cu BNP sensing platform toward Cr(vi) was evaluated in the presence of various potentially interfering metal ions including Cr(iii), Fe(iii), Cu(ii), Zn(ii), Ni(ii), Co(ii), Pb(ii), Cd(ii), Hg(ii), and Mn(ii). All investigated species were tested individually at an identical concentration of 100 µM under the same experimental conditions to enable direct comparison of their colorimetric responses. As shown in Fig. 11, Cr(vi) generated a markedly higher absorbance response at 652.0 nm than the other tested metal ions, whereas the potentially interfering species produced comparatively weak responses.

**Fig. 11:**
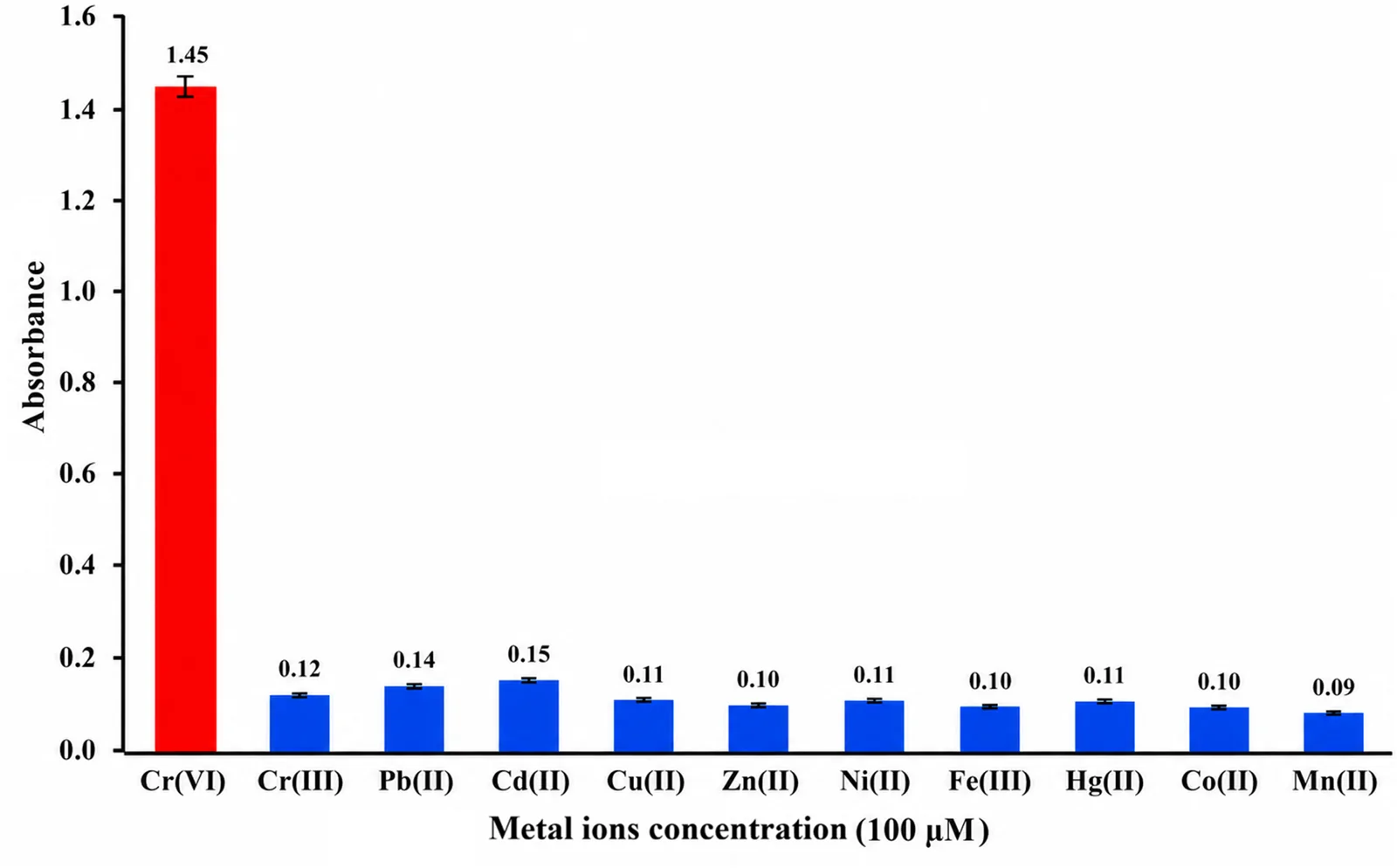
Selectivity evaluation of the Fe–Cu BNP nanozyme platform for Cr(vi) detection. The absorbance responses at 652.0 nm were recorded for Cr(vi) and various potentially interfering metal ions (100 µM each) including Cr(iii), Fe(iii), Cu(ii), Zn(ii), Ni(ii), Co(ii), Pb(ii), Cd(ii), Hg(ii), and Mn(ii), under identical experimental conditions. The higher response obtained for Cr(vi) compared with the investigated interfering ions demonstrates the favorable selectivity of the proposed colorimetric sensing platform under the investigated conditions. Error bars represent the standard deviation of three independent experimental determinations (*n* = 3).

The pronounced response toward Cr(vi) demonstrates the favorable selectivity of the developed sensing system under the experimental conditions investigated. In particular, the substantially lower response observed for Cr(iii) compared with Cr(vi) indicates that the oxidation state of chromium strongly influences the colorimetric response of the TMB-based sensing system. Similarly, the limited responses produced by the other investigated metal ions indicate relatively low interference with Cr(vi) detection when examined individually at the same concentration.

These findings demonstrate that the proposed Fe–Cu BNP/TMB sensing system possesses good selectivity and anti-interference capability toward Cr(vi) under the investigated conditions. Together with the concentration-dependent response and excellent linearity observed for Cr(vi), the selectivity results support the potential applicability of the developed platform for environmental monitoring. However, because real environmental water samples were not investigated in the present study, further validation using representative environmental matrices is required to establish the accuracy, precision, recovery, and matrix tolerance of the proposed method under practical environmental-analysis conditions.

### 3.11. Validation using biological samples

The practical applicability of the proposed GOx/Fe–Cu BNP sensing platform was further evaluated through glucose determination in six independent human serum samples. The glucose concentrations obtained using the proposed method showed good agreement with the corresponding values measured using the commercial glucose assay kit as summarized in Table 5. The glucose concentrations determined by the proposed method ranged from 3.91 to 7.10 mM demonstrating the applicability of the developed sensing platform across different serum glucose levels.

**Table 5:** Determination of glucose in six independent human serum samples using the proposed GOx/Fe–Cu BNP sensing platform and comparison with a commercial glucose assay kit

Sample	Reference method (mM)	Proposed method (mM)	Relative error (%)	Glucose added (mM)	Glucose found (mM)	Recovery (%)	RSD (%)
S1	4.96	5.08 ± 0.11	+2.42	1.00	6.07 ± 0.08	99.0	1.32
S2	4.42	4.47 ± 0.07	+1.13	2.00	6.51 ± 0.09	102.0	1.38
S3	5.38	5.31 ± 0.08	−1.30	4.00	9.35 ± 0.13	101.0	1.39
S4	6.15	6.09 ± 0.09	−0.98	1.00	7.08 ± 0.10	99.0	1.41
S5	3.87	3.91 ± 0.06	+1.03	2.00	5.95 ± 0.08	102.0	1.34
S6	7.02	7.10 ± 0.10	+1.14	4.00	11.06 ± 0.15	99.0	1.36

The accuracy of the proposed method in the serum matrix was further assessed through standard-addition recovery experiments. Satisfactory recoveries ranging from 99.0 to 102.0% were obtained with RSD values below 1.5% demonstrating good accuracy and precision and indicating negligible matrix interference. Furthermore, statistical comparison of the results obtained using the proposed method and the commercial glucose meter was performed using a paired Student's *t*-test at the 95% confidence level. The calculated *t*-value was 0.856 with a corresponding *p*-value of 0.431. Since *p* > 0.05, no statistically significant difference was observed between the two methods. These findings confirm the satisfactory accuracy, precision, and practical applicability of the GOx/Fe–Cu BNP sensing platform for glucose determination in human serum samples.

### 3.12. Stability, reproducibility, and sustainability assessment

The storage stability and reproducibility of the Fe–Cu BNP nanozyme platform were quantitatively evaluated under the optimized experimental conditions. The catalytic activity of the Fe–Cu BNPs was monitored over a 30-day storage period by measuring the TMB/H_2_O_2_ response at 652.0 nm. The nanozyme retained 91.8% of its initial catalytic activity after 30 days demonstrating satisfactory storage stability. Reproducibility was further evaluated using three independent experimental determinations (*n* = 3), yielding an RSD of 1.6% which demonstrates satisfactory precision and reproducibility of the developed sensing platform.

The sustainability, practical applicability, and analytical performance of the proposed platform were systematically evaluated using the NAT,^52^ BAGI,^53^ RAPI,^54^ and EPPI^55^ assessment metrics with the overall outcomes summarized in Fig. 12. The NAT assessment yielded an overall score of 75, reflecting the combined contributions of the different assessment criteria. This overall score should not be interpreted as indicating that all components employed during synthesis are environmentally benign. The contribution of thiourea should also be taken into account in the overall sustainability assessment of the developed platform. The less favorable synthesis-related criteria observed in the NAT assessment further indicate that these aspects are incorporated into the overall evaluation.

**Fig. 12:**
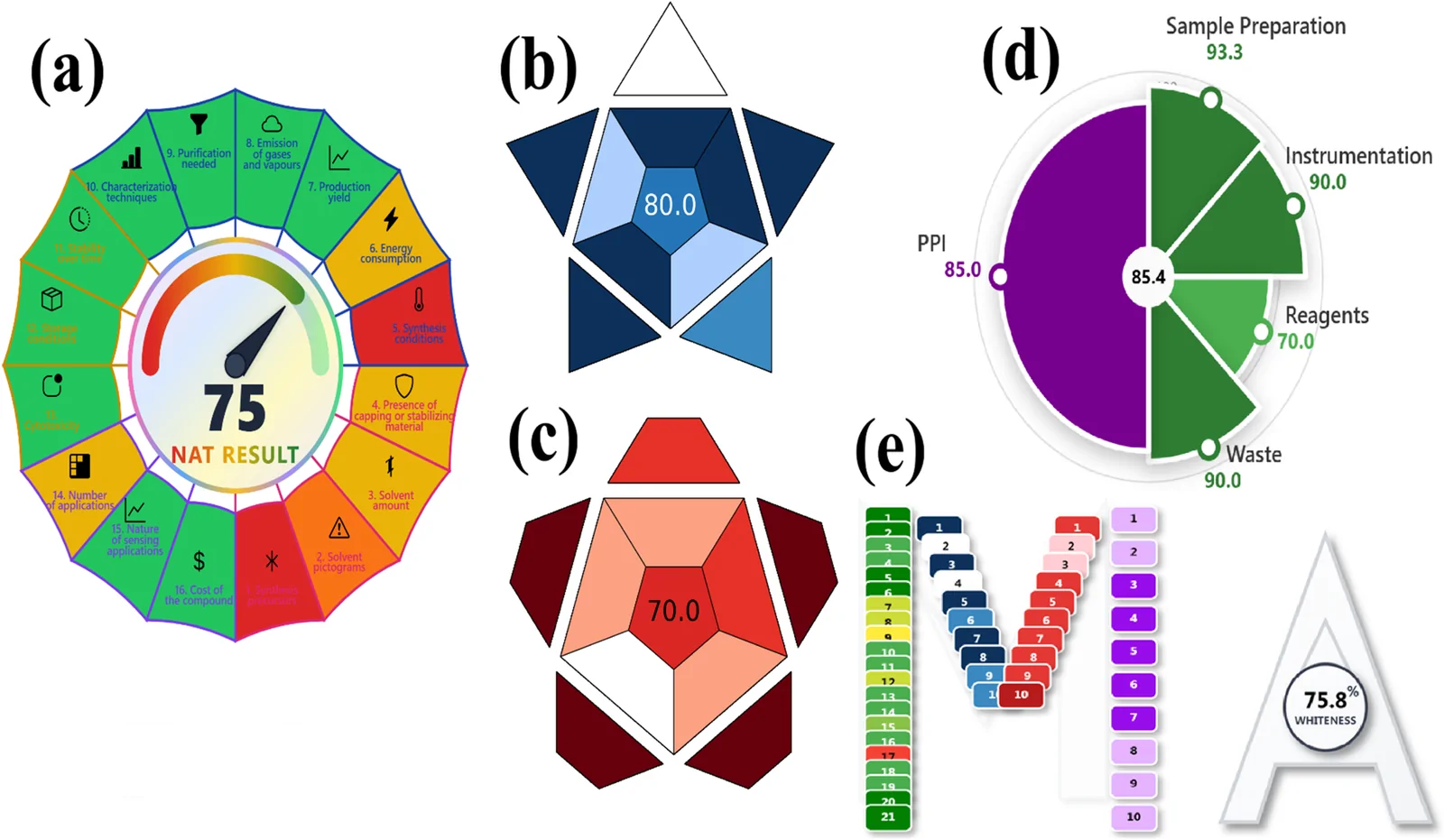
Sustainability, applicability, and analytical performance assessment of the developed Fe–Cu BNP-based sensing platform. (a) Nanomaterial Assessment Tool (NAT) evaluation showing an overall score of 75. (b) Blue Applicability Grade Index (BAGI) assessment yielding a score of 80.0. (c) Red Analytical Performance Index (RAPI) assessment yielding a score of 70.0. (d) Environmental Performance-Practicality Index (EPPI) assessment showing an overall score of 85.4, with individual scores of 93.3 for sample preparation, 90.0 for instrumentation, 70.0 for reagents, 90.0 for waste, and 85.0 for the practicality performance index (PPI). (e) Integrated assessment showing an overall whiteness score of 75.8%, reflecting the balance among analytical performance, environmental considerations, and practical applicability.

The BAGI assessment yielded an overall score of 80, indicating favorable practical applicability of the proposed analytical platform, whereas the RAPI score of 70 demonstrated satisfactory analytical performance. The EPPI evaluation generated an overall score of 85.4, reflecting the combined environmental and practical characteristics of the developed methodology. The individual EPPI components were 93.3 for sample preparation, 90.0 for instrumentation, 70.0 for reagents, 90.0 for waste, and 85.0 for the practicality performance index (PPI). Notably, the comparatively lower reagent score of 70.0 reflects reagent-related safety and environmental considerations and provides a balanced representation of the overall sustainability profile.

Furthermore, the integrated assessment yielded an overall whiteness score of 75.8%, indicating a favorable balance among analytical performance, environmental considerations, and practical applicability. This score also indicates opportunities for further improvement of the sustainability profile particularly through the future consideration of safer synthesis reagents while maintaining the analytical efficiency and practical advantages of the developed platform. The complete NAT, BAGI, RAPI, and EPPI assessment criteria and their corresponding scores are provided in Table S1 of the SI.

## 4. Conclusion

This study presents an eco-conscious Fe–Cu bimetallic nanozyme platform integrating aqueous nanomaterial synthesis, peroxidase-mimicking catalysis, and multimodal colorimetric detection. Structural characterization confirmed the formation of nanoscale Fe–Cu-containing structures with particle sizes ranging from approximately 6.49 to 16.30 nm and an average diameter of 11.7 nm. The Fe–Cu BNPs effectively catalyzed TMB oxidation in the presence of H_2_O_2_ with the optimum catalytic response obtained at pH 4.0, 40 °C, and an incubation time of 10 min. Steady-state kinetic analysis yielded a *K*_m_ value of 0.18 mM and a *V*_max_ of 8.92 × 10^−8^ M s^−1^ supporting favorable substrate affinity and catalytic activity.

The developed platform enabled quantitative H_2_O_2_ determination over 10–700 µM and was successfully coupled with GOx for glucose determination over 10–500 µM. Spectrophotometric, Arduino-assisted, and smartphone-based measurements produced linear responses with coefficients of determination above 0.99 demonstrating consistency among the three analytical approaches. Practical applicability was demonstrated through glucose determination in six human serum samples with recoveries of 99.0–102.0% and RSD values below 1.5%. Statistical comparison with the reference method showed no significant difference (*p* = 0.431). The Fe–Cu BNP platform also provided a concentration-dependent response toward Cr(vi) over 0–500 µM with *R*^2^ = 0.9991 and favorable selectivity against the investigated interfering metal ions. Control experiments indicated that Cr(vi) directly oxidizes TMB, while Fe–Cu BNPs enhance the resulting optical response; therefore, the nanozyme is considered a signal-amplifying component of the Cr(vi) sensing system. Further validation using real environmental samples is required to establish its performance in complex environmental matrices.

The developed nanozyme retained 91.8% of its initial catalytic activity after 30 days supporting its storage stability. Sustainability evaluation yielded NAT, BAGI, RAPI, and EPPI scores of 75, 80, 70, and 85.4, respectively. Overall, the integration of eco-conscious synthesis, peroxidase-mimicking catalysis, quantitative colorimetric sensing, portable smartphone- and Arduino-assisted detection, and sustainability assessment demonstrates the potential of the Fe–Cu BNP platform for decentralized glucose analysis and future environmental sensing applications.

## Ethical statement

All experimental procedures involving human serum samples were approved by the Ethics Committee of Future University in Egypt and conducted in accordance with the ethical principles of the World Medical Association Declaration of Helsinki. Human serum samples were purchased under formal authorization from VACSERA (The Holding Company for Biological Products and Vaccines, Giza, Egypt). Informed consent was obtained from all human participants by the providing institution.

## Conflicts of interest

There are no conflicts to declare.

## Supplementary Material

RA-016-D6RA07383B-s001

## Data Availability

The authors confirm that the data supporting the findings of this study are available within the article. Supplementary information (SI) is available. See DOI: https://doi.org/10.1039/d6ra07383b.

## References

[cit1] Haghayegh F., Norouziazad A., Haghani E., Feygin A. A., Rahimi R. H., Ghavamabadi H. A., Sadighbayan D., Madhoun F., Papagelis M., Felfeli T. (2024). Adv. Sci..

[cit2] Plebani M., Nichols J. H., Luppa P. B., Greene D., Sciacovelli L., Shaw J., Khan A. I., Carraro P., Freckmann G., Dimech W. (2025). Clin. Chem. Lab. Med..

[cit3] Yang S.-M., Lv S., Zhang W., Cui Y. (2022). Sensors.

[cit4] Sacks D. B., Bruns D. E., Goldstein D. E., Maclaren N. K., McDonald J. M., Parrott M. (2002). Clin. Chem..

[cit5] Sacks D. B., Arnold M., Bakris G. L., Bruns D. E., Horvath A. R., Kirkman M. S., Lernmark A., Metzger B. E., Nathan D. M. (2011). Clin. Chem..

[cit6] Rajendran S. , Porwal A., Anjali K., Anvaya and Anuradha R., Internet of Things in Bioelectronics: Emerging Technologies and Applications, 2024, pp. 263–296

[cit7] Sher M., Zhuang R., Demirci U., Asghar W. (2017). Expert Rev. Mol. Diagn..

[cit8] Wu J., Wang X., Wang Q., Lou Z., Li S., Zhu Y., Qin L., Wei H. (2019). Chem. Soc. Rev..

[cit9] Huang Y., Ren J., Qu X. (2019). Chem. Rev..

[cit10] Singh S. (2019). Front. Chem..

[cit11] Hussein O. G., Abdelkawy M. (2025). Microchem. J..

[cit12] Hussein O. G., Naguib I. A., Althobaiti S. A., Elsayed M. A., Gamal M. (2026). Microchem. J..

[cit13] Abdelgawad M. A., Sanari J. A., Gamal M., Hussein O. G. (2026). RSC Adv..

[cit14] Kulandaivel S., Lin C.-H., Yeh Y.-C. (2022). Chem. Commun..

[cit15] Kulandaivel S., Chen H.-T., Lin C.-H., Yeh Y.-C. (2023). J. Mater. Chem. B.

[cit16] Zhu X., Zheng L., Zhao P., Gao L., Wang L., Liu J., Yang X., Wei H., Zhang M., Yan L. (2025). Adv. Healthcare Mater..

[cit17] Sherazee M., Ahmed S. R., Das P., Srinivasan S., Rajabzadeh A. R. (2023). Colloids Surf., A.

[cit18] Hassan H. A., Sedky N. K., Nafie M. S., Mahdy N. K., Fawzy I. M., Fayed T. W., Preis E., Bakowsky U., Fahmy S. A. (2024). Molecules.

[cit19] Chi Z., Wang Q., Gu J. (2023). Analyst.

[cit20] Kurup C. P., Ahmed M. U. (2023). Biosensors.

[cit21] Hussein O. G., Naguib I. A., Bayram R., Alluhayb A. H., Ali R., Gamal M. (2026). Anal. Methods.

[cit22] Pietrzak M., Ivanova P. (2021). Sens. Actuators, B.

[cit23] Sun X., Cui J., Tan X., Zhen Z., Tan W., Gao X., Yu K., Li G., Chai H., Zhang G. (2026). Small.

[cit24] Gawande M. B., Goswami A., Felpin F.-X., Asefa T., Huang X., Silva R., Zou X., Zboril R., Varma R. S. (2016). Chem. Rev..

[cit25] Li X., You J., Li J., Wang Z., Zhao Y., Xu J., Duan M., Zhang H. (2024). ChemCatChem.

[cit26] Cao S., Long Y., Xiao S., Deng Y., Ma L., Adeli M., Qiu L., Cheng C., Zhao C. (2023). Chem. Soc. Rev..

[cit27] Li L., Cao L., Xiang X., Wu X., Ma L., Chen F., Cao S., Cheng C., Deng D., Qiu L. (2022). Adv. Funct. Mater..

[cit28] Kirubakaran D., Wahid J. B. A., Karmegam N., Jeevika R., Sellapillai L., Rajkumar M., SenthilKumar K. (2026). Biomed. Mater. & Devices.

[cit29] Ijaz I., Gilani E., Nazir A., Bukhari A. (2020). Green Chem. Lett. Rev..

[cit30] Samuel M. S., Ravikumar M., John J A., Selvarajan E., Patel H., Chander P. S., Soundarya J., Vuppala S., Balaji R., Chandrasekar N. (2022). Catalysts.

[cit31] Shreyash N., Bajpai S., Khan M. A., Vijay Y., Tiwary S. K., Sonker M. (2021). ACS Appl. Nano Mater..

[cit32] Li S., Zhang Y., Wang Q., Lin A., Wei H. (2021). Anal. Chem..

[cit33] Fuente-Ballesteros A., Samanidou V., Ares A. M., Bernal J. (2025). Sustainable Chem. Pharm..

[cit34] Rahman M. S., Hiers W., Davidson A., Chabot C., Riley F., Coutant M. (2025). J. Pharm. Sci..

[cit35] Pingili D., Awasthi A., Varshney M. M. (2025). J. Anal. Chem..

[cit36] Tovar-Lopez F. J. (2023). Sensors.

[cit37] Pal P. , Pandey B., Prakash A., Sarangi P. K., Rathore S. S., Agarwal V., Gupta A. K. and Singh A. K., in Biotechnology for Environmental Sustainability, Springer, 2025, pp. 561–591

[cit38] Sharma P., Singh S. P., Parakh S. K., Tong Y. W. (2022). Bioengineered.

[cit39] Saha R., Nandi R., Saha B. (2011). J. Coord. Chem..

[cit40] Agrawal A., Kumar V., Pandey B. (2006). Miner. Process. Extr. Metall. Rev..

[cit41] Dawra N., Dabas N. (2024). Int. J. Environ. Anal. Chem..

[cit42] Zhang L., Bi X., Liu X., He Y., Li L., You T. (2023). Nanoscale.

[cit43] Yang Z., Guo J., Wang L., Zhang J., Ding L., Liu H., Yu X. (2024). Small.

[cit44] Hossain M. A., Canning J. (2024). Adv. Devices Instrum..

[cit45] Fan K., Liu W., Miao Y., Li Z., Liu G. (2023). Adv. Intell. Syst..

[cit46] Fan Y., Li J., Guo Y., Xie L., Zhang G. (2021). Measurement.

[cit47] Tsebesebe N. T., Mpofu K., Sivarasu S., Mthunzi-Kufa P. (2025). Discov. Internet Things.

[cit48] Sahara F. A., Sultana M. S., Amin M. K., Shamim Al Mamun M., Dhar P. K., Dutta S. K. (2025). ChemistryOpen.

[cit49] Gao L., Zhuang J., Nie L., Zhang J., Zhang Y., Gu N., Wang T., Feng J., Yang D., Perrett S. (2007). Nat. Nanotechnol..

[cit50] Filippova A. D., Sozarukova M. M., Baranchikov A. E., Kottsov S. Y., Cherednichenko K. A., Ivanov V. K. (2023). Molecules.

[cit51] Huang F., Wang J., Chen W., Wan Y., Wang X., Cai N., Liu J., Yu F. (2018). J. Taiwan Inst. Chem. Eng..

[cit52] Abbas A. E. F., Magdy G., Halim M. K. (2025). Microchem. J..

[cit53] Manousi N., Wojnowski W., Płotka-Wasylka J., Samanidou V. (2023). Green Chem..

[cit54] Nowak P. M., Wojnowski W., Manousi N., Samanidou V., Płotka-Wasylka J. (2025). Green Chem..

[cit55] Elagamy S. H., Kannaiah K. K. P., Chanduluru H. K., Yahaya N., Obaydo R. H. (2025). Green Anal. Chem..

