# Supplementary material for: Eco-conscious Fe–Cu bimetallic nanozymes as a multimodal colorimetric platform for glucose and hexavalent chromium determination
Source: RSC Adv. 2026 Sep 29;16(45):58447–66. doi: 10.1039/d6ra07383b (PMC13621409; doi:10.1039/d6ra07383b)
Supplement: Supplementary file 1 [file RA-016-D6RA07383B-s001.pdf]

Supplementary Information (SI) for RSC Advances.
This journal is © The Royal Society of Chemistry

Supplementary Information
For

Eco-Conscious Fe-Cu Bimetallic Nanozymes as a Multimodal Colorimetric Platform for Glucose and Hexavalent Chromium Determination

Saja A. Althobaiti[1], Ola G. Hussein[2]*

[1] *Department of Chemistry, College of Science and Humanities in Al-Kharj, Prince Sattam Bin Abdulaziz University, Al-Kharj 11942, Saudi Arabia.*

[2] *Department of Pharmaceutical Chemistry, Faculty of Pharmacy, Future University in Egypt, Cairo, 11835, Egypt*

*Corresponding author email: ola.farag@fue.edu.eg

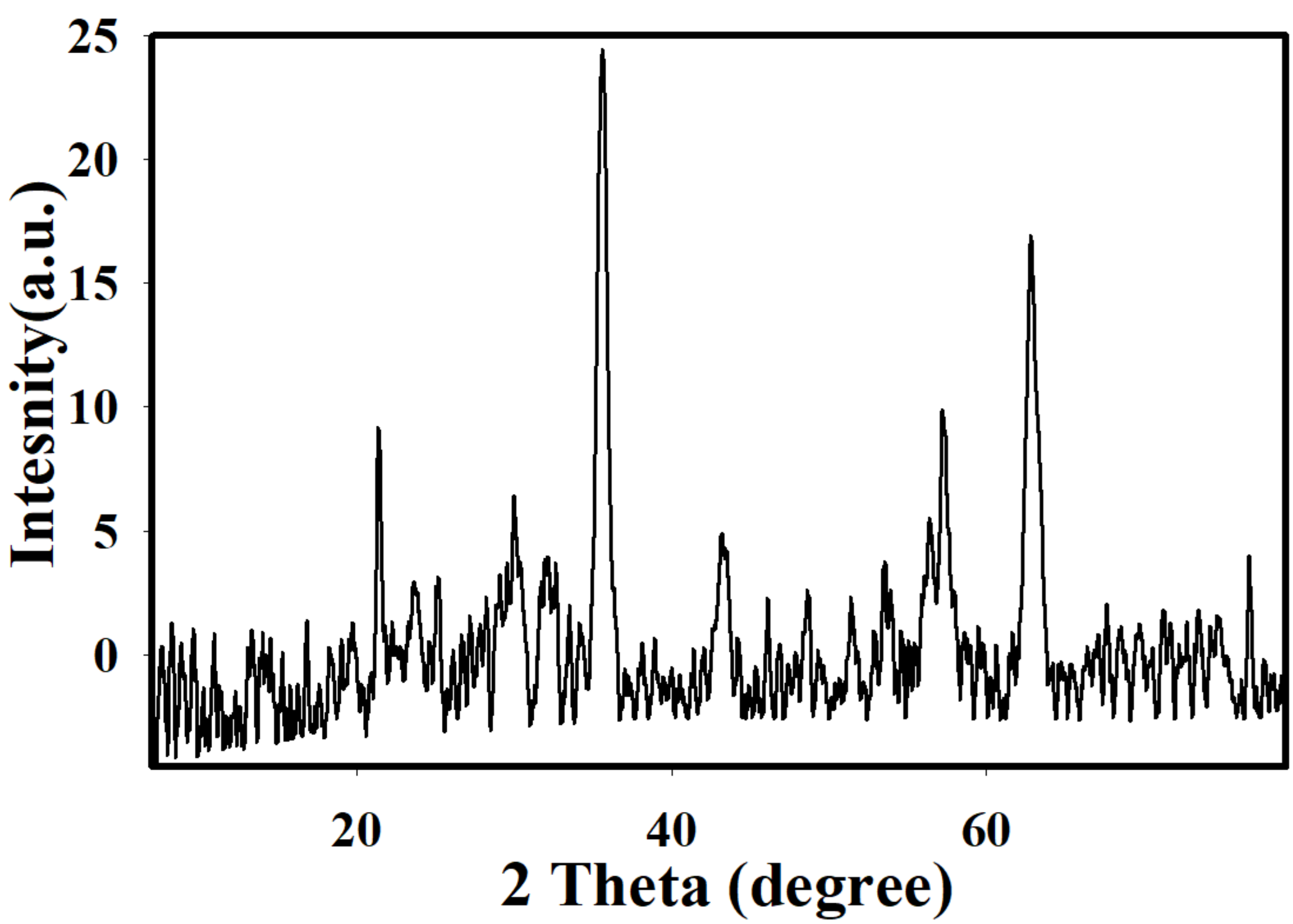

Fig. S1 XRD pattern of the synthesized Fe-Cu BNPs showing characteristic diffraction peaks at approximately 35°, 57°, and 63° (2θ), indexed to the (211), (321), and (224) crystallographic planes, respectively, consistent with the $CuFe_2O_4$ reference pattern (JCPDS No. 34-0425).

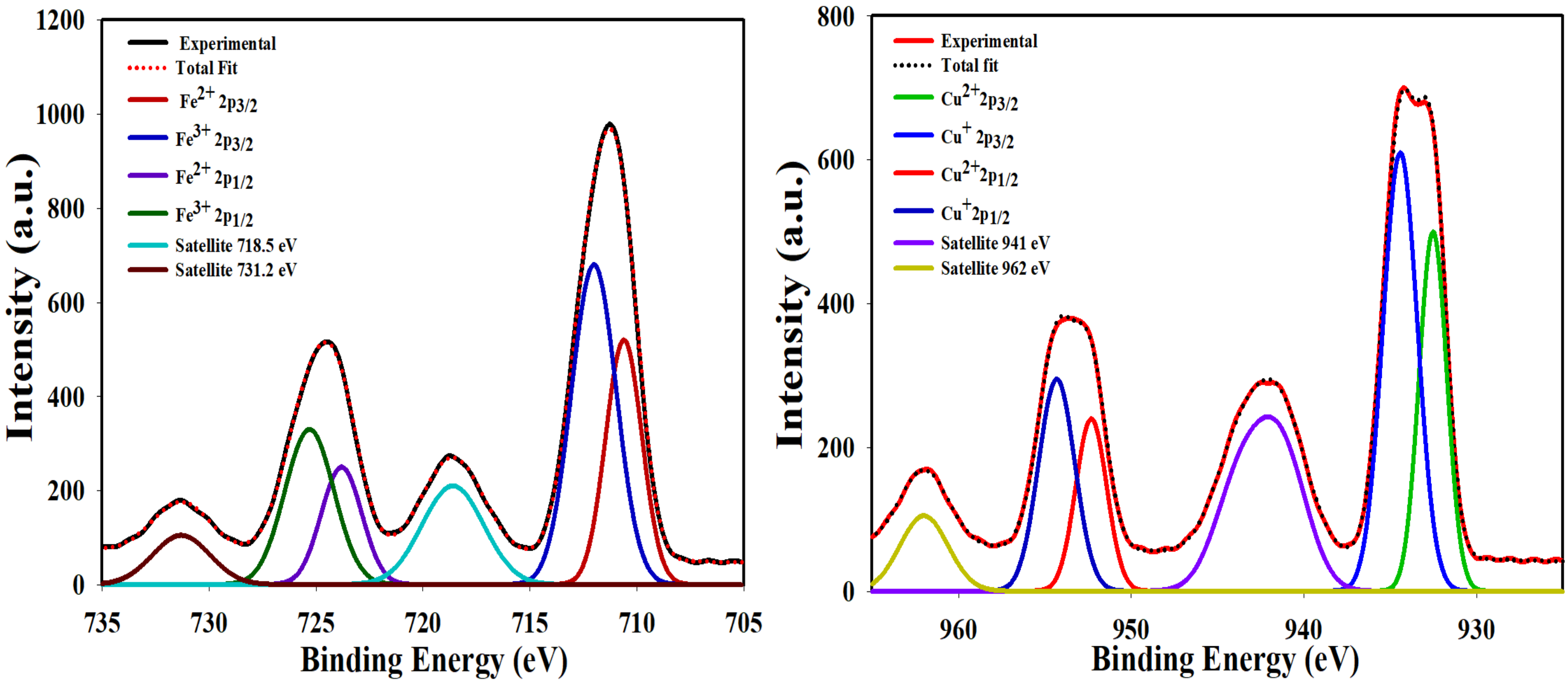

Fig. S2 High-resolution XPS spectra of the synthesized Fe-Cu BNPs: (a) Fe 2p spectrum showing the deconvoluted Fe^{2+} and Fe^{3+} components and corresponding satellite features; and (b) Cu 2p spectrum showing Cu^{+} and Cu^{2+} components together with characteristic satellite peaks.

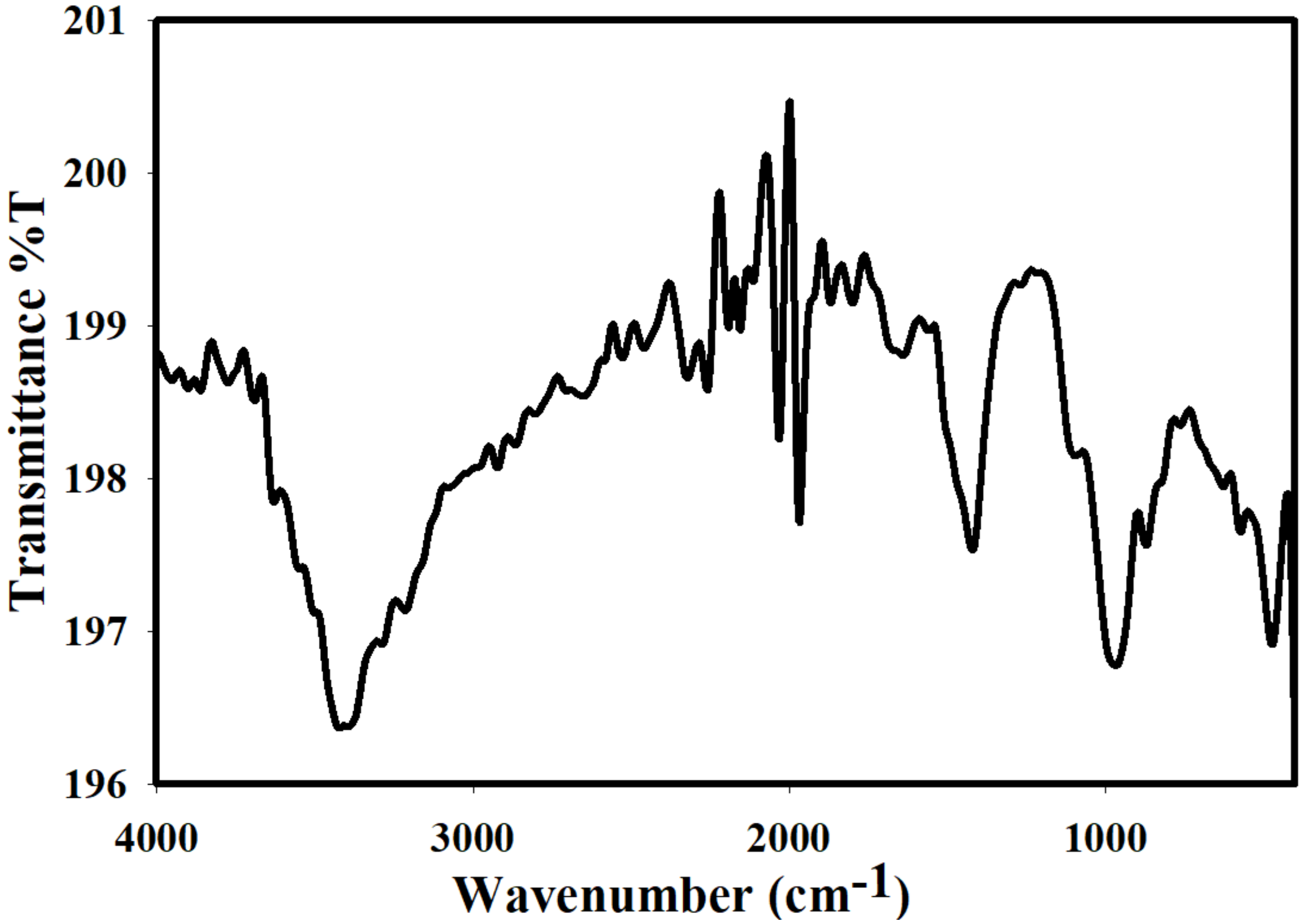

Fig. S3 FTIR spectrum of the synthesized Fe-Cu bimetallic nanozymes (Fe-Cu BNPs) recorded in the range of 4000-500 cm^{-1}. The spectrum exhibits characteristic absorption bands corresponding to surface hydroxyl groups, residual organic moieties from the synthesis process and metal-oxygen vibrations associated with the formation of Fe–O and Cu–O bonds confirming the successful synthesis and stabilization of the Fe-Cu bimetallic nanostructure.

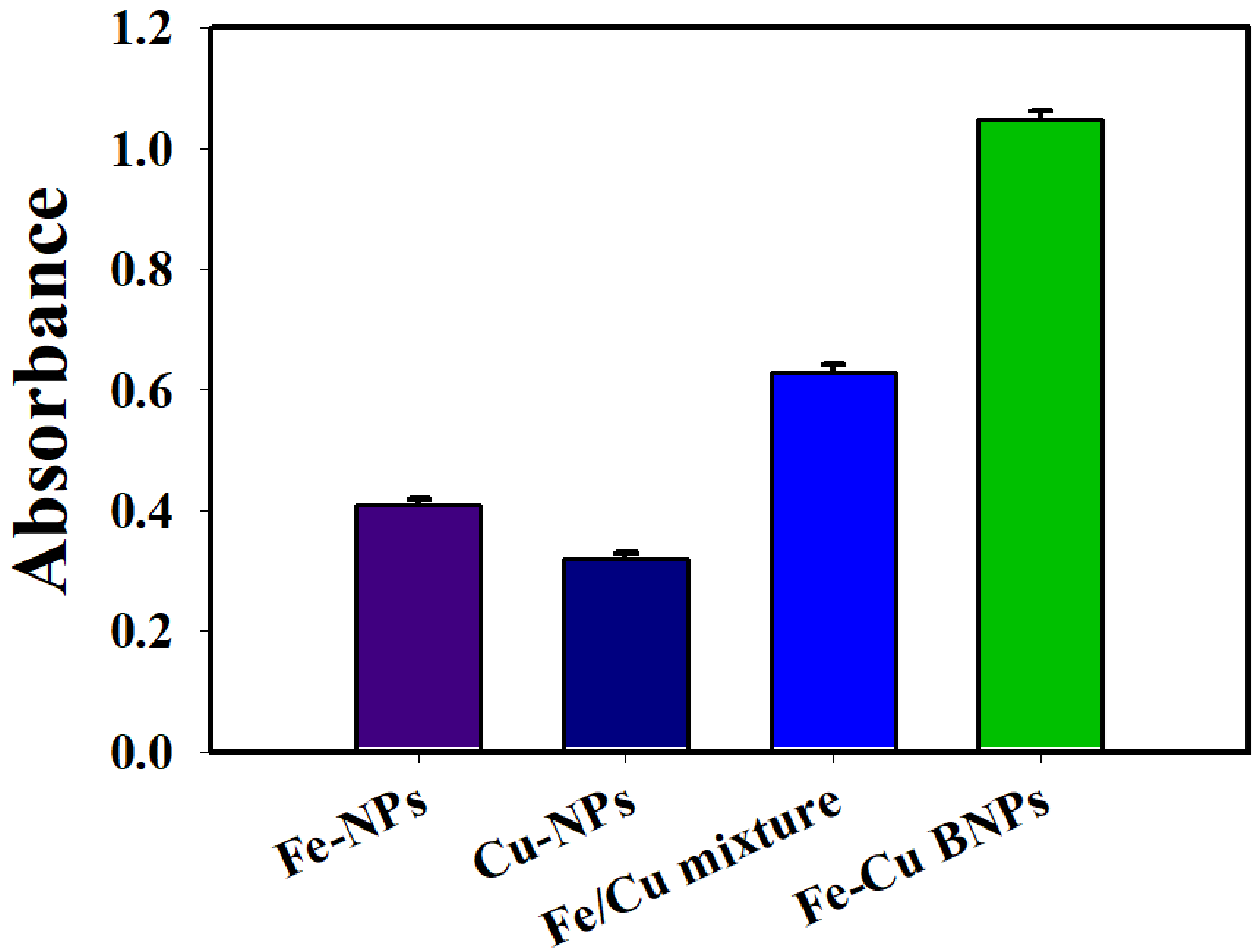

Fig. S4 Comparative peroxidase-like catalytic activity of Fe-only NPs, Cu-only NPs, a physical Fe/Cu mixture, and Fe-Cu BNPs toward the TMB/H_2O_2 system. Catalytic activity was evaluated by measuring the absorbance of oxTMB at 652.0 nm under identical experimental conditions. The Fe-Cu BNPs exhibited a higher catalytic response than the corresponding monometallic nanoparticles and their physical mixture. Data are expressed as mean ± SD (n = 3).

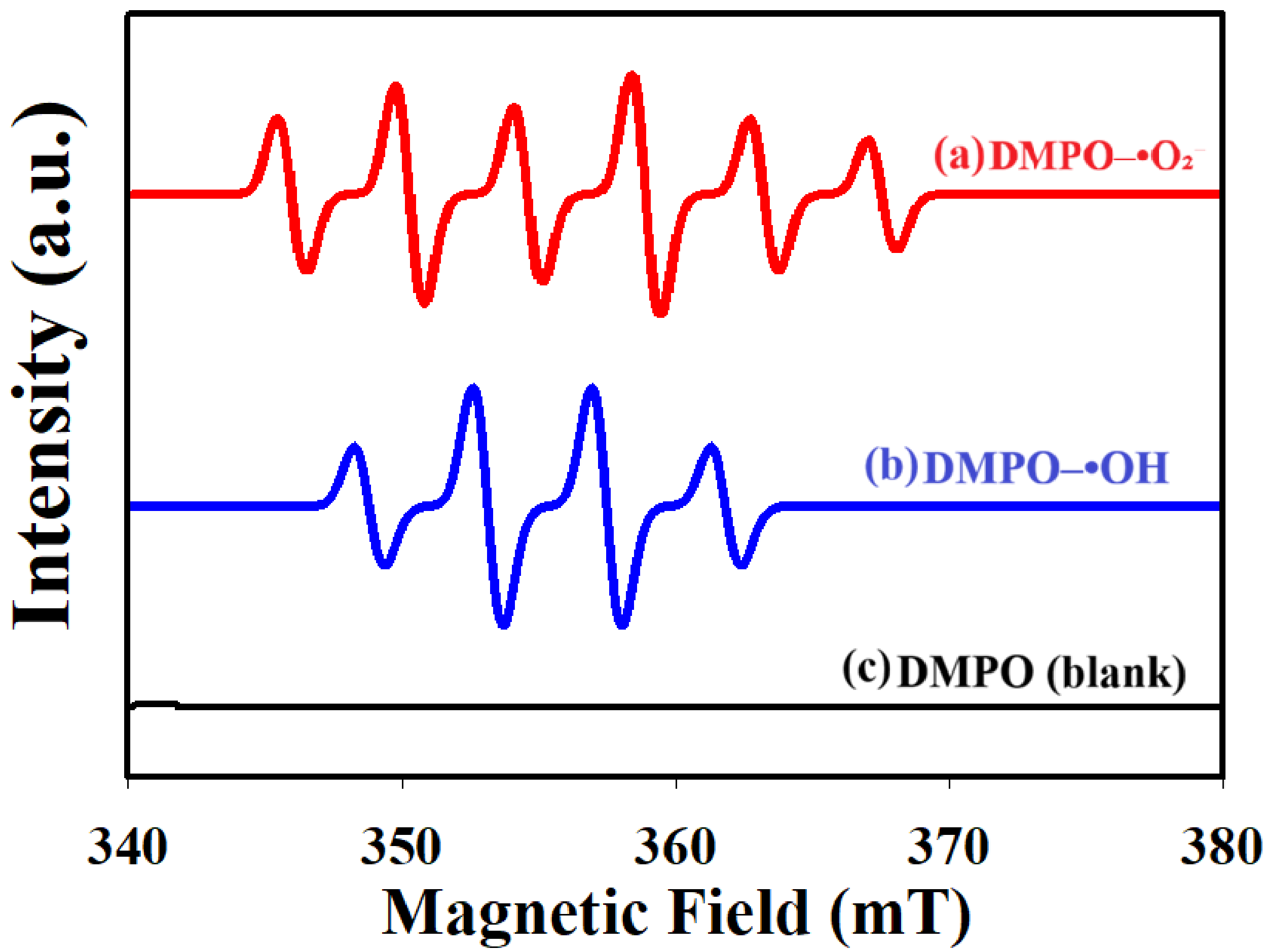

Fig. S5 EPR spin-trapping spectra of reactive oxygen species generated in the Fe-Cu BNP/ H_2O_2 catalytic system using DMPO as the spin-trapping agent. Signals assigned to the DMPO–•OH and DMPO–•O₂⁻-derived spin adducts were observed, whereas the DMPO blank showed no appreciable EPR signal. These results support the generation of hydroxyl- and superoxide-derived reactive oxygen species during the Fe-Cu BNP-mediated peroxidase-like catalytic reaction.

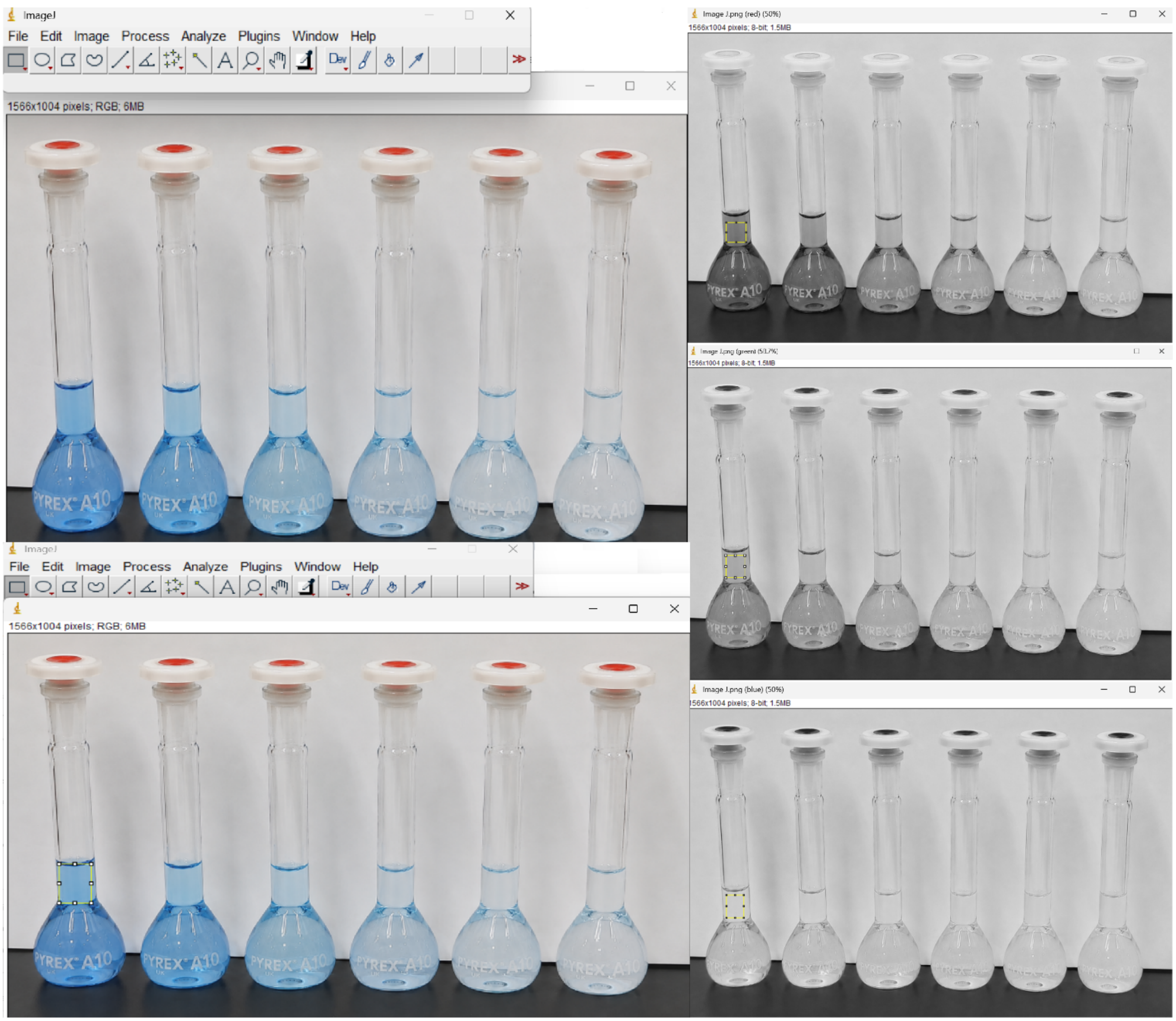

ImageJ
File Edit Image Process Analyze Plugins Window Help
1566x1004 pixels; RGB; 6MB
PYREX A10
Image J.png (red) (50%)
1566x1004 pixels; 8-bit; 1.5MB
Image J.png (blue) (50%)
1566x1004 pixels; 8-bit; 1.5MB

Fig. S6 Representative smartphone-assisted digital image analysis of the Fe-Cu BNP colorimetric sensing system. The original colorimetric image acquired using an iPhone 15 Pro and its subsequent ImageJ processing are shown including ROI selection and separation into the red, green, and blue channels. An identical ROI was applied to all samples, and the mean red-channel intensity was used for quantitative analysis.

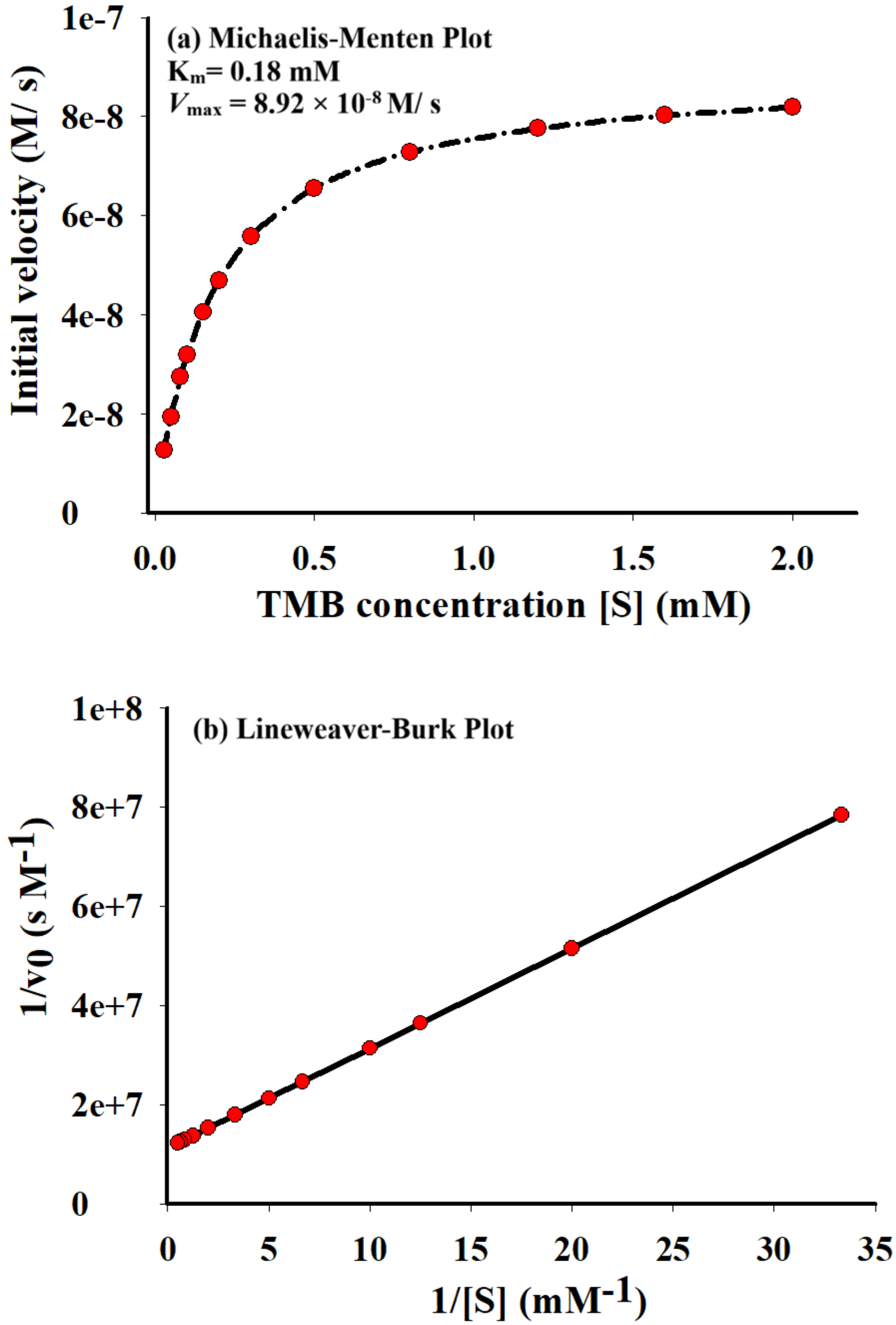

Fig. S7 Steady-state kinetic analysis of the peroxidase-like activity of Fe-Cu BNPs toward TMB. (a) Michaelis-Menten plot of the initial reaction velocity (v_0) as a function of TMB concentration [S]. (b) Corresponding Lineweaver-Burk plot of $1/v_0$ versus 1/[S]. The kinetic parameters were determined as K_m of 0.18 mM and V_{max} of 8.92×10^{-8} M/ s.

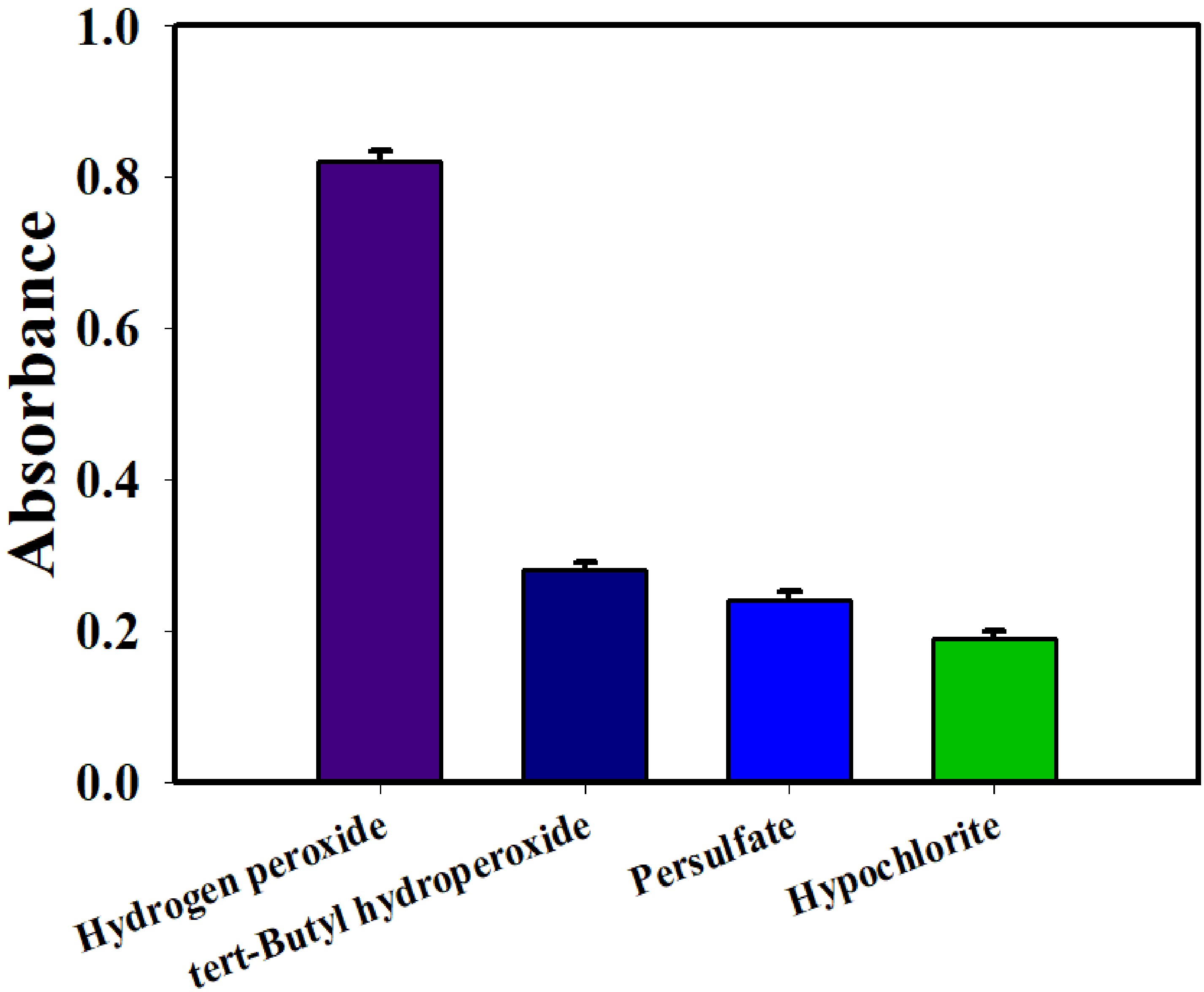

Fig. S8 Selectivity of the Fe-Cu BNP/TMB catalytic system toward different oxidizing species. The colorimetric responses toward hydrogen peroxide (H_2O_2), tert-butyl hydroperoxide (TBHP), persulfate ($S_2O_8^{2-}$), and hypochlorite (ClO^-) were measured at 652.0 nm under identical experimental conditions. Data are presented as mean ± SD (n = 3).

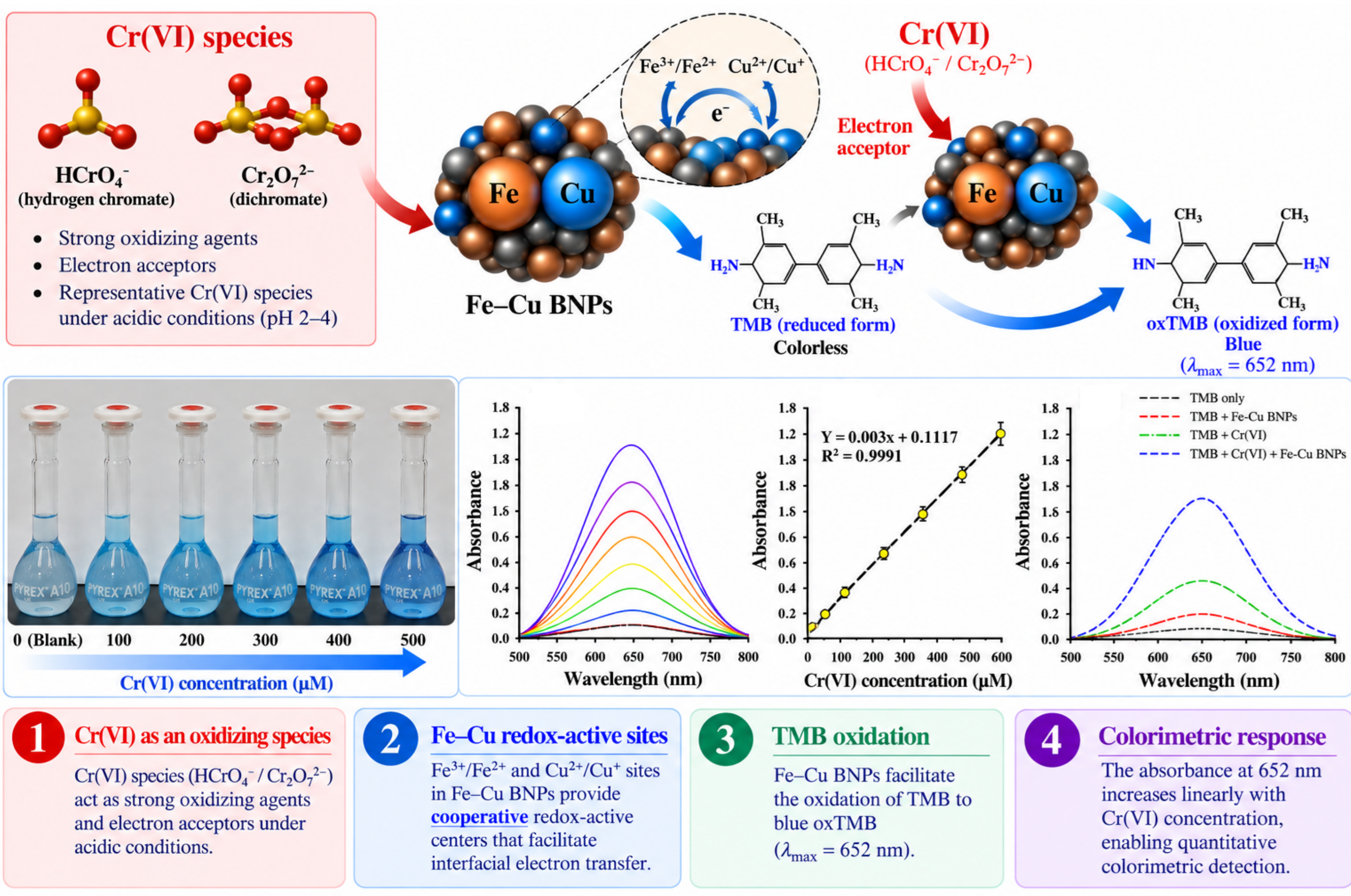

Fig. S9 Proposed mechanism for the colorimetric sensing of Cr(VI) using the Fe-Cu BNP/TMB system. Cr(VI) species act as oxidizing agents and electron acceptors under acidic conditions, while the cooperative Fe^{3+}/Fe^{2+} and Cu^{2+}/Cu^{+} redox-active sites of the Fe-Cu BNPs facilitate interfacial electron transfer and the oxidation of colorless TMB to blue oxTMB. The resulting formation of oxTMB produces a concentration-dependent increase in absorbance at 652.0 nm enabling quantitative colorimetric determination of Cr(VI).

Table S1. Summary of NAT, BAGI, RAPI, EPPI, and whiteness assessments of the developed Fe-Cu BNP-based sensing platform.

Assessment	Main evaluated aspect	Score	Interpretation
NAT	Nanomaterial sustainability, including synthesis conditions, reagents, energy, purification, stability, and applications	75	Overall sustainability profile; synthesis- and reagent-related concerns, including thiourea, are reflected in the assessment.
BAGI	Practical applicability of the analytical procedure	80.0	Favorable practical applicability.
RAPI	Analytical performance and validation characteristics	70.0	Satisfactory analytical performance.
EPPI	Environmental performance and practicality	85.4	Favorable overall environmental-practical profile; the reagent component was comparatively lower (70.0).
Whiteness	Integrated balance of analytical performance, environmental considerations, and practicality	75.8%	Favorable overall balance with scope for further improvement.
